# Recombinant SADS-CoV as a vector for porcine epidemic diarrhea vaccine development

**DOI:** 10.3389/fimmu.2025.1633661

**Published:** 2025-08-06

**Authors:** Xiaoling Yan, Xiaoli Zhang, Xiaocheng Lyu, Yaoyao Zheng, Qianniu Li, Xiaoya Zhao, Jun Fu, Jingyun Ma

**Affiliations:** ^1^ State Key Laboratory of Swine and Poultry Breeding Industry, College of Animal Science, South China Agricultural University, Guangzhou, China; ^2^ Research and Development Department, ArtemisShield Animal Health Co., Ltd., Guangzhou, China; ^3^ State Key Laboratory of Microbial Technology, Shandong University–Helmholtz Institute of Biotechnology, Shandong University, Qingdao, China

**Keywords:** swine acute diarrhea syndrome coronavirus, reverse genetics, attenuation, viral vector vaccines, porcine epidemic diarrhea virus (PEDV)

## Abstract

**Introduction:**

Swine acute diarrhea syndrome coronavirus (SADS-CoV) is an emerging porcine enteric coronavirus that can cause diarrhea in piglets younger than 5 days of age. However, infection of pigs older than 5 days of age does not usually result in obvious clinical symptoms. This relative intrinsic safety in older animals prompted us to investigate the potential of SADS-CoV as a viral vector for porcine diarrhea virus vaccines.

**Methods:**

We utilized Gibson assembly to clone the SADS-CoV sequence into an artificial bacterial chromosome (BAC) vector. Further manipulation was carried out by recombineering to generate four attenuated recombinant SADS-CoV strains expressing a PEDV protective antigen fused with peptides that target immune cells. Subsequently, the biological characteristics and immunogenic efficacy of these four recombinant strains were systematically assessed through *in vitro* cell models and *in vivo* animal challenge experiments.

**Results and discussion:**

The recombinant viruses exhibited a proliferation profile similar to that of the wild-type virus in Vero cells, maintained stable cytopathic effects, retained the exogenous sequences for up to 20 passages, and consistently expressed the PEDV antigen fusion protein. Immunizing pregnant sows with these recombinant viruses effectively enhanced both cellular and mucosal immune responses and provided significant clinical protection against PEDV to their offspring. This study not only generated a vaccine candidate for PEDV but also established a pipeline for using the SADS-CoV as a vector for vaccine development.

## 1 Introduction

Swine acute diarrhea syndrome coronavirus (SADS-CoV), a novel porcine enteric coronavirus, is an enveloped single-stranded positive-sense RNA virus belonging to the Alphacoronavirus genus of the Coronaviridae family (1). The SADS-CoV genome encodes nonstructural and structural proteins (2), with accessory proteins (NS3a/NS7a/NS7b) being non-essential for viral replication *in vitro* (3–5). This genetic plasticity enables their replacement with exogenous antigens for vector development.

The porcine epidemic diarrhea virus (PEDV) infects pigs of all ages. The clinical symptoms vary depending on the virulence of the strain, the immune status of the herd, and environmental conditions. In neonatal piglets less than two weeks old, infection typically results in acute watery diarrhea, dehydration, vomiting with yellow vomitus, severe wasting, and mortality rate as high as 95% to 100% (6–8). In contrast, adult pigs have relatively lower mortality rates post-infection, although they suffer from symptoms such as watery diarrhea, agalactia, anorexia, depression, and impaired reproductive functions (9, 10). Highly pathogenic PEDV strains lead to growth retardation in weaned piglets, with significantly reduced herd productivity resulting from decreased sow nurturing ability, reduced numbers of live piglets per litter, and lower farrowing rates (11). In comparison, SADS-CoV is less virulent than PEDV, causing diarrhea only in piglets under five days old while older piglets typically do not exhibit obvious clinical symptoms (12). Hence, as a less-pathogenic virus, SADS-CoV has superior biosafety potential as a replicating viral vector for vaccine development.

From 1984 to October 2010, epidemic strains of PEDV in China belonging to the GI subtype caused distinct regional epidemics with relatively low mortality rates in piglets that could be effectively controlled by using inactivated or attenuated live vaccines (13). However, in October 2010, a highly pathogenic PEDV strain emerged in southern China, causing a widespread outbreak of epidemic diarrhea in pigs and rapidly spreading nationwide (14). In some pig farms, the mortality rates of vaccine-immunized piglets were up to 100% (15, 16). Eventually, the transmission of this highly pathogenic GII subtype PEDV strain in China made the GI subtype of strains relatively rare (17). The GI and GII subtypes of PEDV strains have notable genetic differences, particularly in the host receptor-binding protein, which led to deviations in neutralizing antibodies. Therefore, the previous traditional vaccine strains for subtype GI could no longer provide effective protection against infections with strains of the GII subtype (18). Furthermore, commercially available PEDV vaccines, primarily inactivated and live attenuated types, face challenges in balancing safety and efficacy. Inactivated vaccines require high antigen content and intact structure, but the inactivation process can damage antigens and they poorly induce mucosal immunity, resulting in suboptimal protection. Live attenuated vaccines carry risks of virulence reversion and prolonged viral shedding post-immunization, increasing environmental viral load and posing safety concerns (19). Consequently, developing new vaccines that will control PEDV strains of the GII subtype is urgent in the agricultural industry.

The spike (S) protein of PEDV is a type I glycoprotein with 1383 amino acids, including a signal peptide (aa 1-18); neutralizing epitopes (aa 499-638, 748-755, 764-771, and 1368-1374); a transmembrane domain (aa 1334-1356); and a short cytoplasmic domain (20). The core neutralizing epitope region COE of the S1 subunit has been extensively used for the development of subunit vaccines to prevent PEDV infections (21, 22). The S1D (aa 636-789) region was also identified as an epitope region of the PEDV S1 subunit; S1D is highly conserved across PEDV isolates and induces the production of virus neutralization antibodies (23). A multi-epitope vaccine was designed based on the S proteins from the four representative PEDV GII strains (24).

Because PEDV infects intestinal cells, enhancing mucosal immunity is an important consideration in the development of new vaccines. M cells are specialized epithelial cells in gut-associated lymphoid tissues such as Peyer’s patches, isolated lymphoid follicles, the appendix, and other mucosal-associated lymphoid tissue sites, and M cells have been targeted in mucosal vaccine development (25). The specific ligand Co1 for the M cell was identified as a mucosal adjuvant using phage display libraries, and conjugation of Co1 to an antigen results in delivery of the antigen to M cells as well as the induction of Th2-type cytokines (26). Dendritic cells (DCs) are the most common and crucial antigen-presenting cells in gastrointestinal immunity, and they are mostly located beneath the follicle-associated epithelium in the subepithelial dome area where they directly uptake antigens from M cells (27). Molecules that can target and enhance the binding of antigens to DCs are important for improving the efficiency of antigen recognition and uptake by DCs, thereby enhancing the immune response (28). A short peptide consisting of 12 amino acids known as DCpep was identified and used in an innovative immunization strategy in which DCpep, delivered by a *Lactobacillus acidophilus* vector, was employed to transport the protective antigen of *Bacillus anthracis* directly to human DCs (29).

This study explored the potential of SADS-CoV as a viral vector for expressing the PEDV COE and S1D sequences along with the M cell-targeting peptide Co1 and the DC-targeting peptide DCpep. The synergy of cell-targeting peptides and the SADS-CoV viral vector is expected to enhance antigen presentation and the stimulation of mucosal immune responses in the gut. Using Gibson assembly and recombineering for infectious clone generation and manipulation, we present a streamlined approach to develop the SADS-CoV as a novel vector for vaccines.

## 2 Results

### 2.1 Construction of the infectious clone and viral rescue

**Figure 1:**
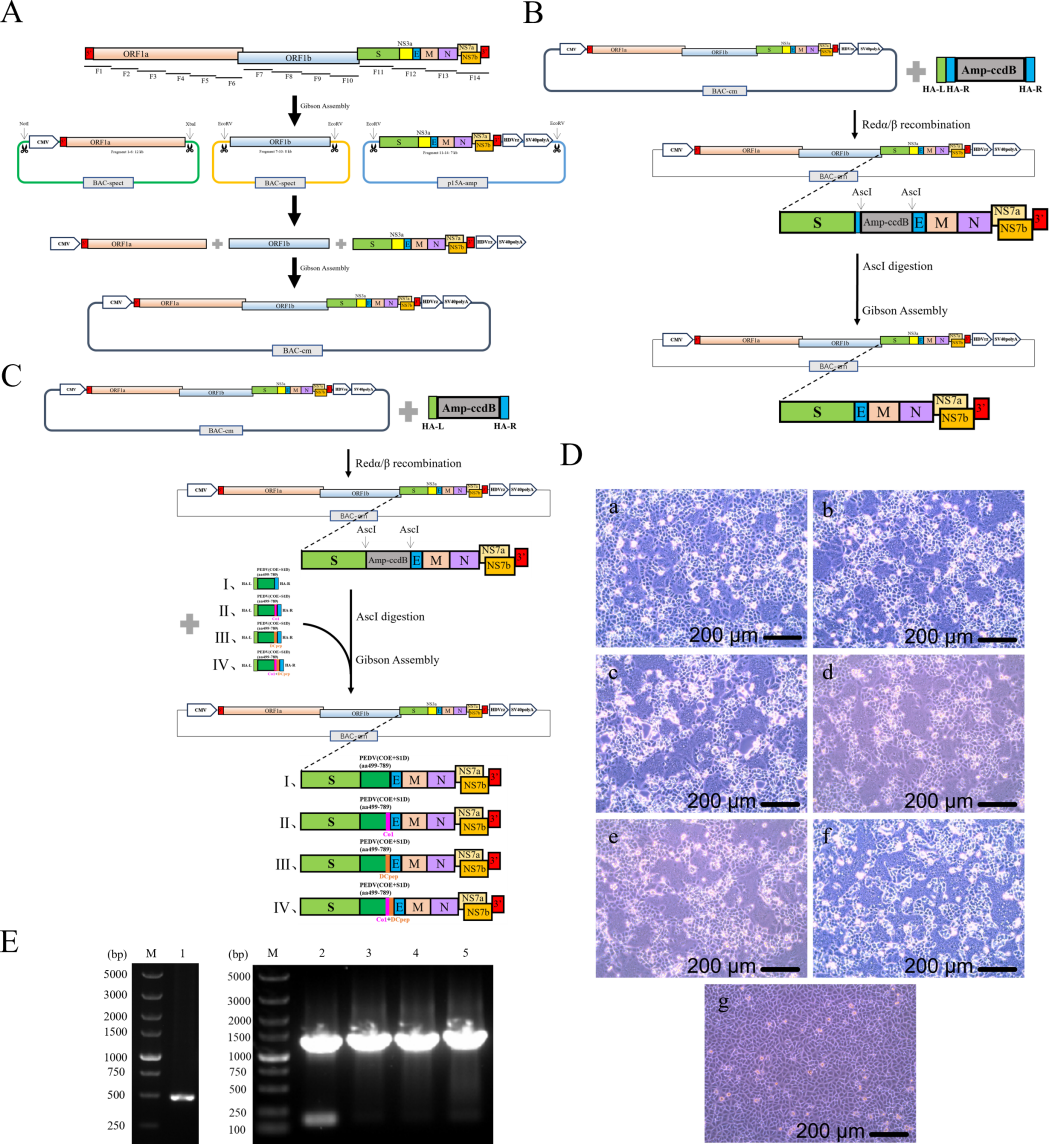
Construction and rescue of rSADS-CoVs. **(A)** Schematic diagram of the construction process for the SADS-CoV full-length infectious clone. F1 to F14 are the overlapping fragments generated by RT-PCR from the SADS-CoV genome and used in Gibson assembly. **(B, C)** Schematic diagrams of the construction of the **(B)** recombinant viral plasmid lacking NS3a and **(C)** recombinant viral plasmids with PEDV antigens, using Redαβ recombineering and Gibson assembly. **(D)** Cytopathic effects produced by the rescued viruses in Vero cells; panel (a), rSADS-ΔNS3a; panel (b), rSADS-ΔNS3a-PEDV(COE+S1D); panel (c), rSADS-ΔNS3a-PEDV(COE+S1D)-Co1; panel (d), rSADS-ΔNS3a-PEDV(COE+S1D)-DCpep; panel (e), rSADS-ΔNS3a-PEDV(COE+S1D)-Co1-DCpep; panel (f), rSADS-CoV; and panel (g), mock infection. **(E)** PCR identification of the rescued viruses by amplification of the NS3a site; lane 1, rSADS-ΔNS3a; lane 2, rSADS-ΔNS3a-PEDV(COE+S1D); lane 3, rSADS-ΔNS3a-PEDV(COE+S1D)-Co1; lane 4, rSADS-ΔNS3a-PEDV(COE+S1D)-DCpep; and lane 5, rSADS-ΔNS3a-PEDV(COE+S1D)-Co1-DCpep. M, marker lanes.

The wild-type infectious clone of SADS-CoV was constructed in two steps (**Figure 1A**), with the first step involving the generation of three intermediate constructs, each containing a different region of the genome. For the first intermediate construct, pBeloBAC11-spect-CI-SADS-CoV-WT-F1-F6, six overlapping linear fragments (F1 to F6) mainly comprising ORF1a were generated by RT-PCR, and then using Gibson assembly, the construct was generated with a spectinomycin-selectable gene and the hCMV promoter. The second intermediate construct, pBeloBAC11-spect-SADS-CoV-WT-F7-F10, was assembled from fragments 7 to 10, which contained the entire ORF1b. The third intermediate construct, p15A-amp-SADS-CoV-WT-F11-F14, contained the remainder of the SADS-CoV sequence on plasmid p15A together with an ampicillin resistance gene, and the hepatitis delta virus ribozyme (HDVrz) and simian virus 40 polyadenylation signal (SV40 polyA) sequences. The second step involved releasing the three large fragments from the intermediate constructs by preparative restriction endoenzyme sites. The released fragments were recombined in a chloramphenicol resistance-conferring artificial bacterial chromosome (BAC) vector by Gibson assembly to obtain the infectious clone pBeloBAC11-cm-CI-SADS-CoV-WT-F1-F14-HDVrz-SV40polyA. The correct construction of the clone was confirmed by restriction enzyme analysis (**Supplementary Figure S1A**).

To generate an infectious deletion mutant for the accessory protein NS3a gene, the PCR-amplified fragment ΔNS3a-amp-ccdB(knockout) was electroporated into *Escherichia coli* GB08-red-gyrA462 containing pBeloBAC11-cm-CI-SADS-CoV-WT-F1-F14-HDVrz-SV40polyA to replace the NS3a coding sequence by lambda Red-mediated recombineering. The resulting plasmid, pBeloBAC11-cm-CI-SADS-CoV-WT-F1-F14-HDVrz-SV40polyA-ΔNS3a-amp-ccdB(knockout), was digested by AscI to remove amp-ccdB and then cyclized by Gibson assembly through the preparative short homologous arms (HA-R). The construct was then transformed into *E. coli* GB2005 to obtain the infectious clone pBeloBAC11-cm-CI-SADS-CoV-WT-F1-F14-HDVrz-SV40polyA-ΔNS3a (**Figure 1B**, **Supplementary Figure S1B**).

To construct the infectious clones for the recombinant SADS-CoV viruses with PEDV antigens, the PCR-amplified fragment ΔNS3a-amp-ccdB(knockin) was electroporated into *E. coli* GB08-red-gyrA462 containing plasmid pBeloBAC11-cm-CI-SADS-CoV-WT-F1-F14-HDVrz-SV40polyA. The intermediate construct pBeloBAC11-cm-CI-SADS-CoV-WT-F1-F14-HDVrz-SV40polyA-ΔNS3a-amp-ccdB(knockin) was then linearized with AscI for incorporation of PEDV(COE+S1D), PEDV(COE+S1D)-Co1, PEDV(COE+S1D)-DCpep, or PEDV(COE+S1D)-Co1-DCpep by Gibson assembly. The constructs were then transformed into *E. coli* GB2005 to obtain the recombinant vectors pBeloBAC11-SADS-CoV-ΔNS3a-PEDV(COE+S1D), pBeloBAC11-SADS-CoV-ΔNS3a-PEDV(COE+S1D)-Co1, pBeloBAC11-SADS-CoV-ΔNS3a-PEDV(COE+S1D)-DCpep, and pBeloBAC11-SADS-CoV-ΔNS3a-PEDV(COE+S1D)-Co1-DCpep (**Figure 1C**, **Supplementary Figure S1C**).

The coding region of the nucleocapsid (N) gene of SADS-CoV was cloned in the pCI-neo vector as a helper plasmid for viral rescue, and Vero cells were then transfected with the various infectious clones of SADS-CoV accompanied with the helper plasmid pCI-SADS-N; the rescued samples exhibited cytopathic effects in the form of syncytia (**Figure 1D**). The rescued recombinant viruses rSADS-ΔNS3a, rSADS-ΔNS3a-PEDV(COE+S1D), rSADS-ΔNS3a-PEDV(COE+S1D)-Co1, rSADS-ΔNS3a-PEDV(COE+S1D)-DCpep, and rSADS-ΔNS3a-PEDV(COE+S1D)-Co1-DCpep were confirmed by PCR amplification around the NS3a site, with results showing the expected bands of 481bp, 1359 bp, 1401 bp, 1401 bp, and 1452 bp, respectively (**Figure 1E**). Sequencing of the PCR products further validated the correct construction of the rescued recombinant SADS-CoV viruses (rSADS-CoVs).

### 2.2 Verification of the attenuation of the NS3a deletion strain

**Figure 2:**
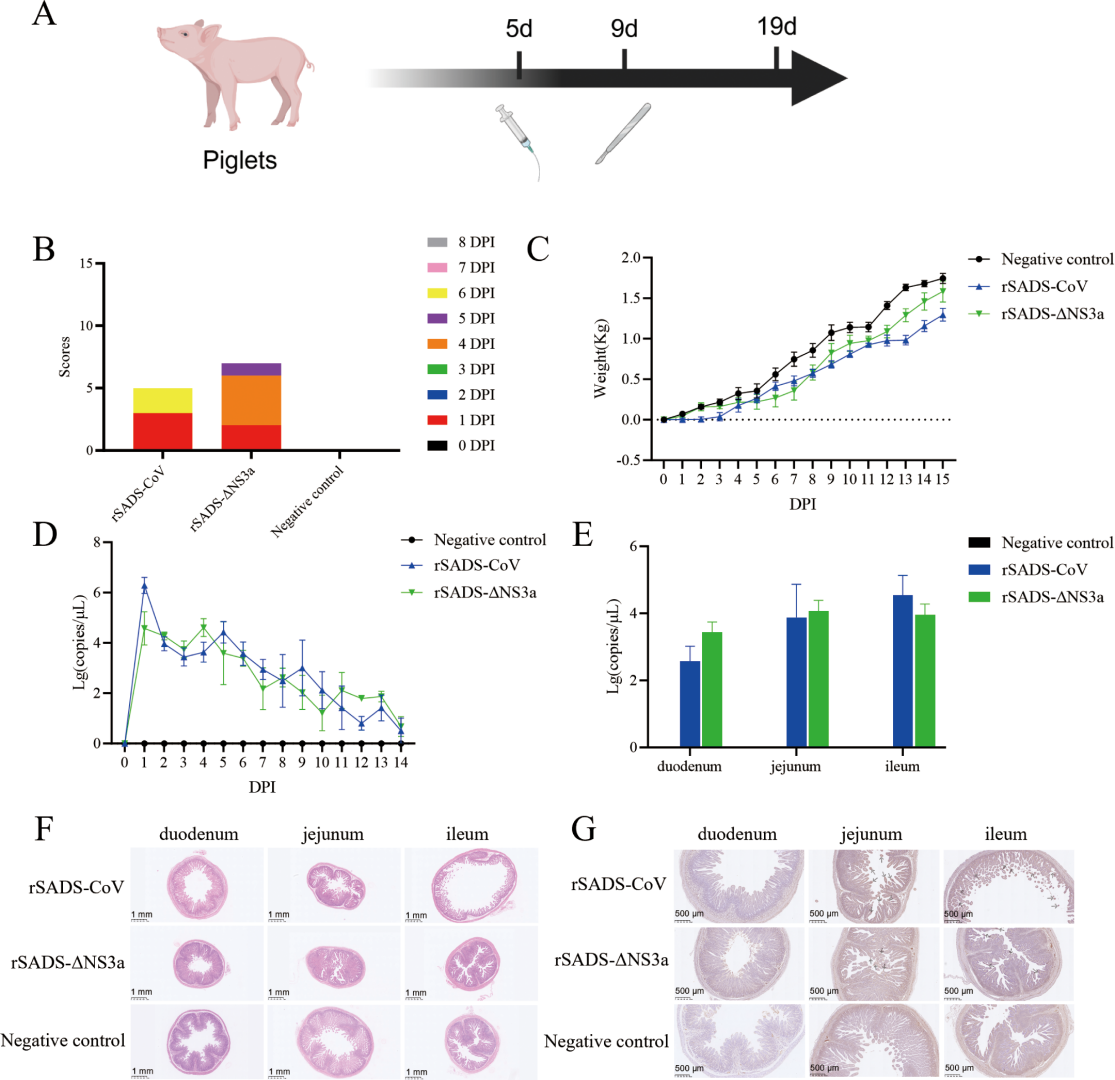
Confirmation of the pathogenicity of SADS-CoV in piglets after deletion of the NS3a accessory protein gene. **(A)** Experimental flowchart; piglets were orally administered with 10 mL of 10^6.8^TCID_50_/mL rSADS-CoV, rSADS-ΔNS3a, or 10 mL DMEM, and three piglets from each group were randomly selected for necropsy on the fourth day following challenge. **(B)** Cumulative clinical diarrhea score. **(C)** Cumulative daily weight gain of piglets after SADS-CoV challenge. **(D)** Viral load in anal swabs of piglets after SADS-CoV challenge. **(E)** Viral copy number in intestinal tissues after SADS-CoV challenge. **(F, G)** Histopathological sections of piglet small intestines were subjected to **(F)** H&E staining to observe tissue damage and **(G)** IHC for detection of SADS-CoV antigen. Arrows indicate areas of more intense DAB staining. Arrows indicate the location of intense DAB staining (30). Note: Some charts contain overlapping data.

The pathogenicity of rSADS-ΔNS3a was compared to that of wild-type rSADS-CoV in animal experiments to determine if the NS3a deletion was attenuating (**Figure 2A**). However, neither the NS3a deletion virus nor the wild-type parental virus caused widespread diarrhea in the piglets after infection; only some piglets exhibited mild-to-moderate diarrhea symptoms, which usually resolved quickly. Consequently, the clinical diarrhea scores were generally low across both groups (**Figure 2B**), making it difficult to compare the pathogenicity of the two virus strains based solely on clinical symptoms. Therefore, other parameters, such as weight gain in the piglets and viral shedding, were assessed.

There were no significant differences in weight gain between the rSADS-ΔNS3a group and the rSADS-CoV group, indicating that the NS3a deletion virus and parental virus had similar impacts on piglet growth (**Figure 2C**). Rectal swab samples were collected daily post-inoculation to extract viral RNA, and absolute quantitative fluorescence was used to measure viral nucleic acid copies. Both infection groups exhibited a peak in viral shedding from days 1 to 4 post-inoculation, with no significant differences in the amount or duration of viral shedding between the rSADS-ΔNS3a and rSADS-CoV groups (**Figure 2D**).

On day 4 post-inoculation, three piglets from each group were randomly selected for necropsy. Results showed that the small intestines of piglets in the negative control group had thick walls, while the rSADS-CoV group exhibited significantly thinner and more transparent intestinal walls, with yellow watery stools. In contrast, the rSADS-ΔNS3a group showed milder intestinal lesions, with only partial segments having thinner and more transparent walls. Thus, the intestinal lesions caused by the accessory protein NS3a deletion virus were less severe than those caused by the parental virus. As shown in **Figure 2E**, no SADS-CoV nucleic acid was detected in the duodenum, jejunum, or ileum of the negative control group. However, in both the rSADS-ΔNS3a and rSADS-CoV groups, virus was detected in the duodenum, jejunum, and ileum, although with no significant differences in overall viral load between the two groups. Histopathological analysis revealed that the two infection groups exhibited varying degrees of small intestinal villus damage compared to the negative control group. The rSADS-CoV group had more severe ileal villus damage, while the rSADS-ΔNS3a group showed only mild villus damage in the small intestine (**Figure 2F**). Therefore, the intestinal tissue damage caused by the NS3a deletion virus was less severe than that caused by the parental virus. The small intestine tissue sections were then subjected to immunohistochemistry (IHC) to detect SADS-CoV N protein. The small intestinal villus epithelial cells of the inoculated piglets were colored to varying degrees by the 3,3’-diaminobenzidine (DAB) chromogen. The rSADS-CoV group showed significant coloration in the jejunum and ileum, while only a small amount of coloration was observed in the duodenum, indicating a higher presence of SADS-CoV antigens in the severely damaged jejunum and ileum. The rSADS-ΔNS3a group showed some coloration in the ileum and jejunum and only a small amount in the duodenum, indicating less presence of SADS-CoV antigens in the jejunum and ileum when compared to the rSADS-CoV group (**Figure 2G**).

### 2.3 Biological characterization of the recombinant viruses with PEDV antigens

**Figure 3:**
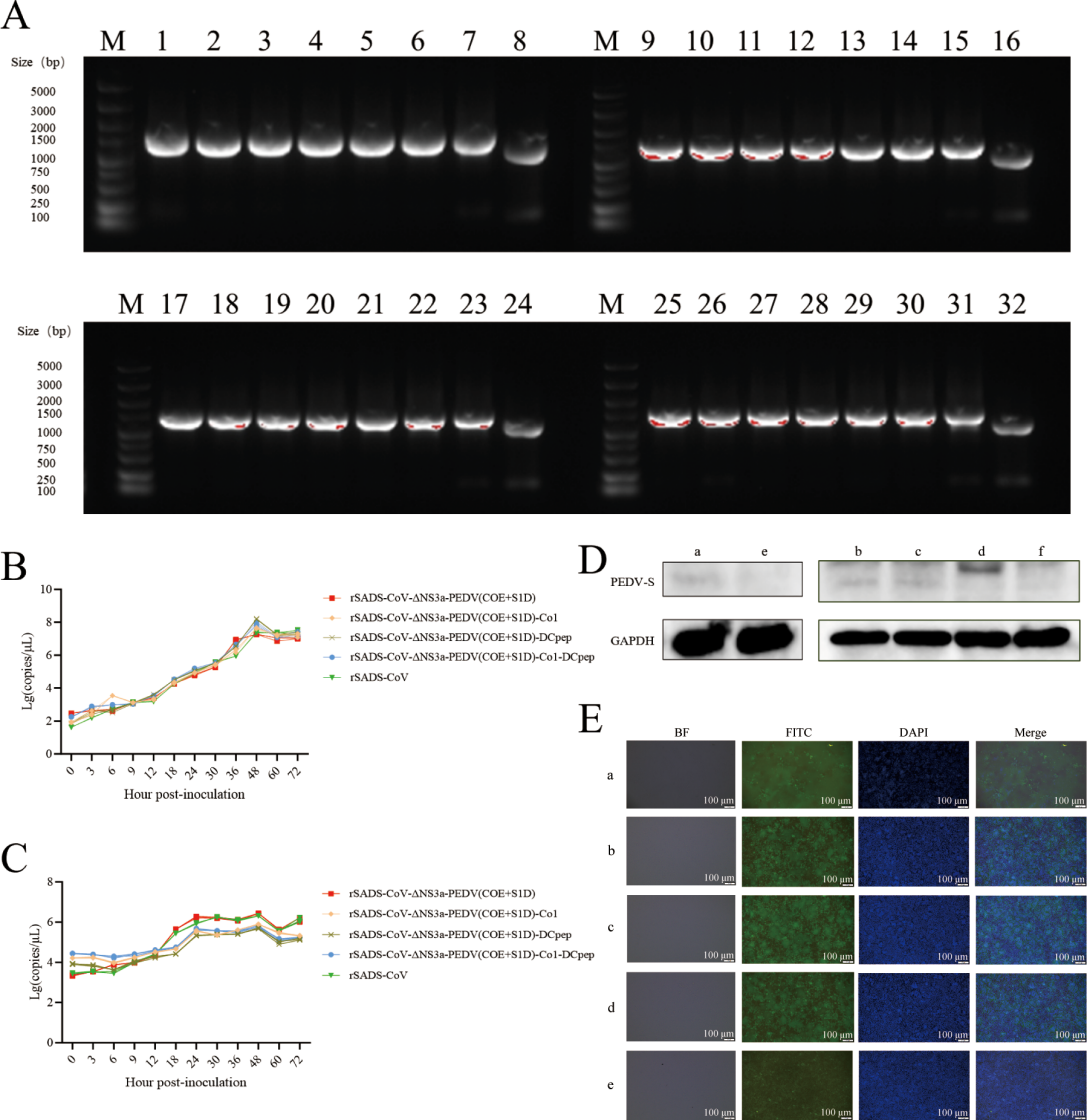
Biological characterization of the recombinant viruses expressing PEDV antigens. **(A)** PCR evaluation of the stability of the inserted PEDV antigen sequences in the recombinant viruses during passage. The PCR was conducted using primers that flank the NS3a site. Lanes 1 to 7, 9 to 15, 17 to 23, and 25 to 31 show results for rSADS-CoV-ΔNS3a-PEDV(COE+S1D), rSADS-CoV-ΔNS3a-PEDV(COE+S1D)-Co1, rSADS-CoV-CMV(T-C)-ΔNS3a-PEDV(COE+S1D)-DCpep, and rSADS-CoV-PEDV(COE+S1D)-Co1-DCpep at passages 2, 5, 8, 11, 14, 17, and 20, respectively. Lanes 8, 16, 24, and 32, rSADS-CoV. **(B, C)** One-step growth curves of recombinant viruses in **(B)** Vero cells and **(C)** IPI-2I cells. **(D)** Evaluation of the presence of PEDV antigen protein by western blot; a, rSADS-CoV-ΔNS3a-PEDV(COE+S1D); b, rSADS-CoV-ΔNS3a-PEDV(COE+S1D)-Co1; c, rSADS-CoV-ΔNS3a-PEDV(COE+S1D)-DCpep; d, rSADS-CoV-ΔNS3a-PEDV(COE+S1D)-Co1-DCpep; e and f, mock-infected cells. **(E)** Identification of PEDV antigen protein by IFA; a, rSADS-CoV-ΔNS3a-PEDV(COE+S1D); b, rSADS-CoV-ΔNS3a-PEDV(COE+S1D)-Co1; c, rSADS-CoV-ΔNS3a-PEDV(COE+S1D)-DCpep; d, rSADS-CoV-ΔNS3a-PEDV(COE+S1D)-Co1-DCpep; and e, mock-infected cells. DAPI staining (blue fluorescence) was used to visualize nuclear DNA, while FITC-conjugated antibodies (green fluorescence) specifically labeled the PEDV COE+S1D antigen.

The recombinant viruses expressing PEDV antigens were inoculated into Vero cells and continuously passaged to the 20th generation, with stable formation of lesions observed throughout the passaging. Maintenance of the exogenous genes was demonstrated by PCR for generations 2, 5, 8, 11, 14, 17, and 20 (**Figure 3A**). Additionally, sequencing of these PCR products confirmed that the amino acid sequences of the inserted fragments had no mutations, and no compensatory mutations occurred in the SADS-CoV vector sequence even after 20 passages (**Supplementary Figure S2**). This indicates that the PEDV antigen gene can be stably maintained on the SADS-CoV vector.

The growth trends of the recombinant viruses rSADS-CoV-ΔNS3a-PEDV(COE+S1D), rSADS-CoV-ΔNS3a-PEDV(COE+S1D)-Co1, rSADS-CoV-ΔNS3a-PEDV(COE+S1D)-DCpep, and rSADS-CoV-ΔNS3a-PEDV(COE+S1D)-Co1-DCpep, alongside the parental virus rSADS-CoV, were assessed in Vero cells. All four recombinant viruses showed proliferation trends similar to the parental virus, reaching peak titers at 48 hours (**Figure 3B**). In the porcine intestinal epithelial cell line IPI-2I, the three recombinant viruses with targeting peptides showed slightly lower viral copy numbers after 12 hours, indicating slightly lower proliferation levels, when compared to the recombinant virus without targeting peptides and the parental virus (**Figure 3C**). However, the overall growth trends of the four recombinant viruses in IPI-2I cells were fundamentally consistent with that of the parental virus.

The expression of PEDV antigens in the recombinant viruses grown in Vero cells was confirmed through western blot analysis (**Figure 3D**), with results showing target bands of the expected sizes using anti-PEDV spike protein mouse polyclonal antibody, indicating normal expression of the exogenous proteins. Additionally, an immunofluorescence assay (IFA) confirmed the expression of the exogenous proteins in Vero cells inoculated with the recombinant viruses, as evident from the significant fluorescence observed, further validating the normal expression of the inserted protein genes (**Figure 3E**).

### 2.4 Immunogenicity assessment of recombinant viruses

**Figure 4:**
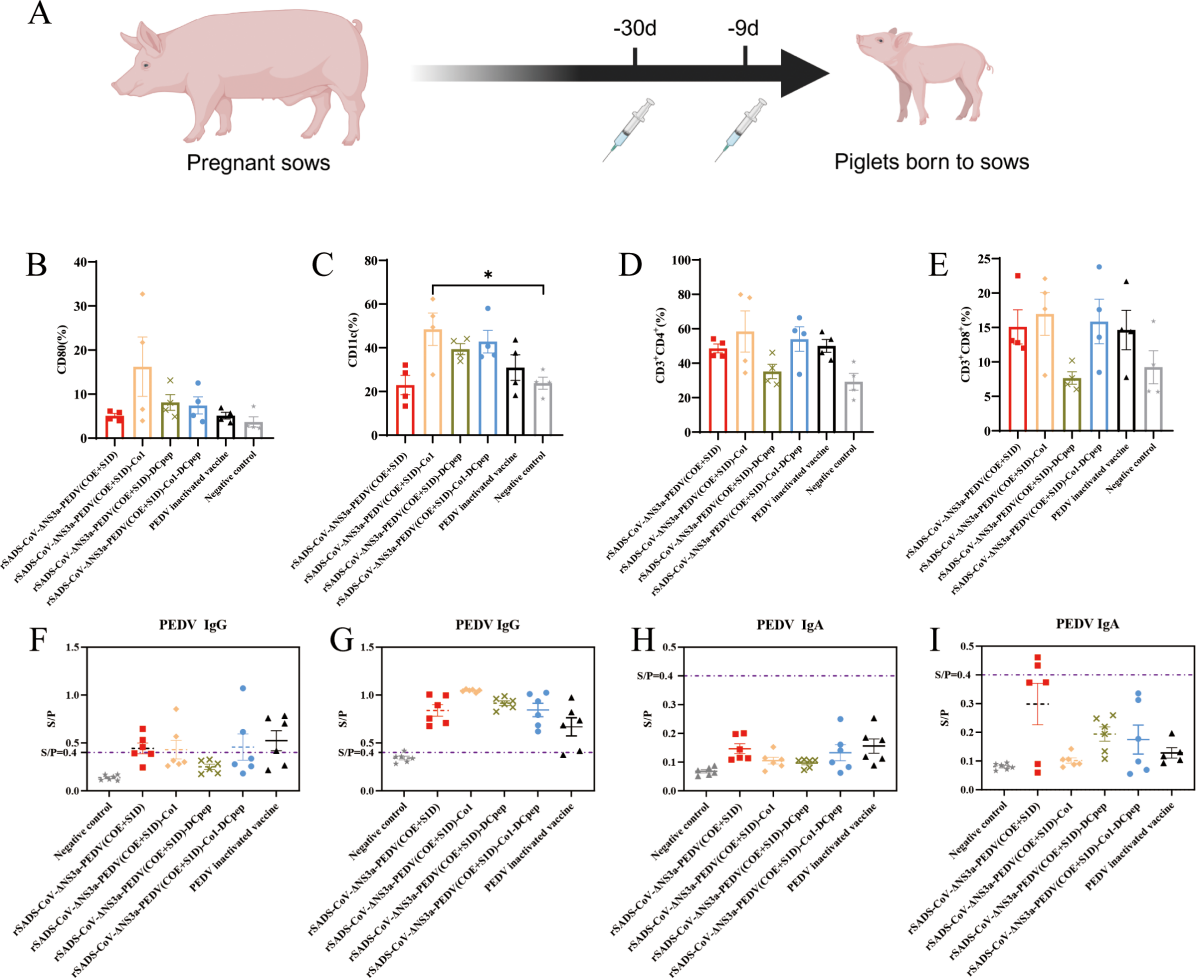
Immunocyte flow cytometry analysis and antibody ELISA detection following immunization of sows with recombinant viruses expressing PEDV antigen. **(A)** Experimental flowchart; pregnant sows were administered with recombinant viruses rSADS-CoV-ΔNS3a-PEDV(COE+S1D), rSADS-CoV-ΔNS3a-PEDV(COE+S1D)-Co1, rSADS-CoV-ΔNS3a-PEDV(COE+S1D)-DCpep, rSADS-CoV-ΔNS3a-PEDV(COE+S1D)-Co1-DCpep, a commercial inactivated PEDV vaccine or DMEM, via Houhai acupoint injections to the sows 30 days before parturition, with a booster given 9 days before delivery. **(B-E)** Peripheral blood of immunized sows was analyzed by flow cytometry for **(B, C)** DC subpopulations of **(B)** CD80 and **(C)** CD11c cells and **(D, E)** T cell subpopulations of **(D)** CD3^+^CD4^+^and **(E)** CD3^+^CD8^+^ cells. *p < 0.05. **(F-I)** The serum of **(F, H)** immunized sows in labor and **(G, I)** the piglets from the immunized sows was analyzed by ELISA for the presence of PEDV S protein-specific **(F, G)** IgG antibodies and **(H, I)** IgA antibodies. S/P= Sample OD/Positive Control OD, positive determination is S/P≥0.4.

The immunogenicity of the recombinant viruses was assessed by inoculating pregnant sows with the viruses (**Figure 4A**, **Table 1**). To evaluate the induction of mucosal and cellular immunity by the recombinant viruses in the sows, peripheral blood was collected at parturition to assess the proportions of DC subgroups CD11c and CD80, and T lymphocyte subgroups CD3^+^CD4^+^ and CD3^+^CD8^+^. The percentages of DCs expressing CD80 in the peripheral blood of sows immunized with rSADS-CoV-ΔNS3a-PEDV(COE+S1D), rSADS-CoV-ΔNS3a-PEDV(COE+S1D)-Co1, rSADS-CoV-ΔNS3a-PEDV(COE+S1D)-DCpep, rSADS-CoV-ΔNS3a-PEDV(COE+S1D)-Co1-DCpep, the inactivated PEDV vaccine, and the negative control group were 5.06 ± 1.08%, 18.32 ± 14.38%, 8.985 ± 4.115%, 8.13 ± 4.37%, 5.215 ± 1.705%, and 4.73 ± 2.47%, respectively (**Figure 4B**, **Supplementary Figures S3G-L**), and the percentages of DCs expressing CD11c were 22.7 ± 9.4%, 44.95 ± 17.35%, 39 ± 5.1%, 46.95 ± 11.05%, 30.9 ± 12.7%, and 23.4 ± 6.7% respectively (**Figure 4C**, **Supplementary Figures S3A-F**). The rSADS-CoV-ΔNS3a-PEDV(COE+S1D)-Co1, rSADS-CoV-ΔNS3a-PEDV(COE+S1D)-DCpep and rSADS-CoV-ΔNS3a-PEDV(COE+S1D)-Co1-DCpep groups exhibited higher proportions of CD80 and CD11c cells when compared to the inactivated vaccine group and the rSADS-CoV-ΔNS3a-PEDV(COE+S1D) group, with the rSADS-CoV-ΔNS3a-PEDV(COE+S1D)-Co1 group showing a significant difference (P ≤ 0.05) in CD11c percentages compared to the negative control group. The percentages of T lymphocyte subgroups CD3^+^CD4^+^ in the immunized and negative control groups were 49.1 ± 5%, 57.15 ± 22.75%, 36.9 ± 9.2%, 49.95 ± 16.45%, 50.05 ± 8.25%, and 29.7 ± 11.2%, respectively (**Figure 4D**, **Supplementary Figures S3M-R**), and the percentages of CD3^+^CD8^+^ were 17.25 ± 5.25%, 15.08 ± 7.02%, 8.12 ± 2.08%, 16.145 ± 7.655, 14.725 ± 6.975%, and 10.78 ± 5.12%, respectively (**Figure 4E**, **Supplementary Figures S3M-R**). The rSADS-CoV-ΔNS3a-PEDV(COE+S1D)-Co1 and rSADS-CoV-ΔNS3a-PEDV(COE+S1D)-Co1-DCpep groups were associated with higher proportions of both CD3^+^CD4^+^ and CD3^+^CD8^+^ cells when compared to the inactivated vaccine group and rSADS-CoV-ΔNS3a-PEDV(COE+S1D) group.

**Table 1:** Immunization grouping and dosing for sows.

Groups	Name of vaccine	Immunization doses	Number of sows	Sow parity
M1	rSADS-CoV-ΔNS3a-PEDV(COE+S1D)	2×10^6.38^TCID_50_/pig	6	1
M2	rSADS-CoV-ΔNS3a-PEDV(COE+S1D)-Co1		6	1
M3	rSADS-CoV-ΔNS3a-PEDV(COE+S1D)-DCpep		6	1
M4	rSADS-CoV-ΔNS3a-PEDV(COE+S1D)-Co1-DCpep		6	1
M5	PEDV inactivated vaccine	2 mL/pig	6	1
M6	DMEM		6	1

To assess the levels of maternally derived antibodies in piglets from the immunized sows, we utilized ELISAs to measure the levels of PEDV S protein-specific IgG and IgA in the serum of sows in labor and piglets prior to challenge. Compared to the negative control, the serum IgG levels in all experimental groups were elevated, with the recombinant virus-immunized groups exhibiting slightly higher overall IgG levels than the inactivated vaccine group (**Figures 4F, G**. Additionally, some piglets in the rSADS-CoV-ΔNS3a-PEDV(COE+S1D) immunized group tested positive for IgA, while IgA levels in other experimental groups did not reach the positive threshold (**Figures 4H, I**). These results indicate that the sows generated antibodies against the PEDV protein and that the antibodies were transferred to the piglets.

### 2.5 Evaluation of the protective effect of recombinant viruses in piglets

**Figure 5:**
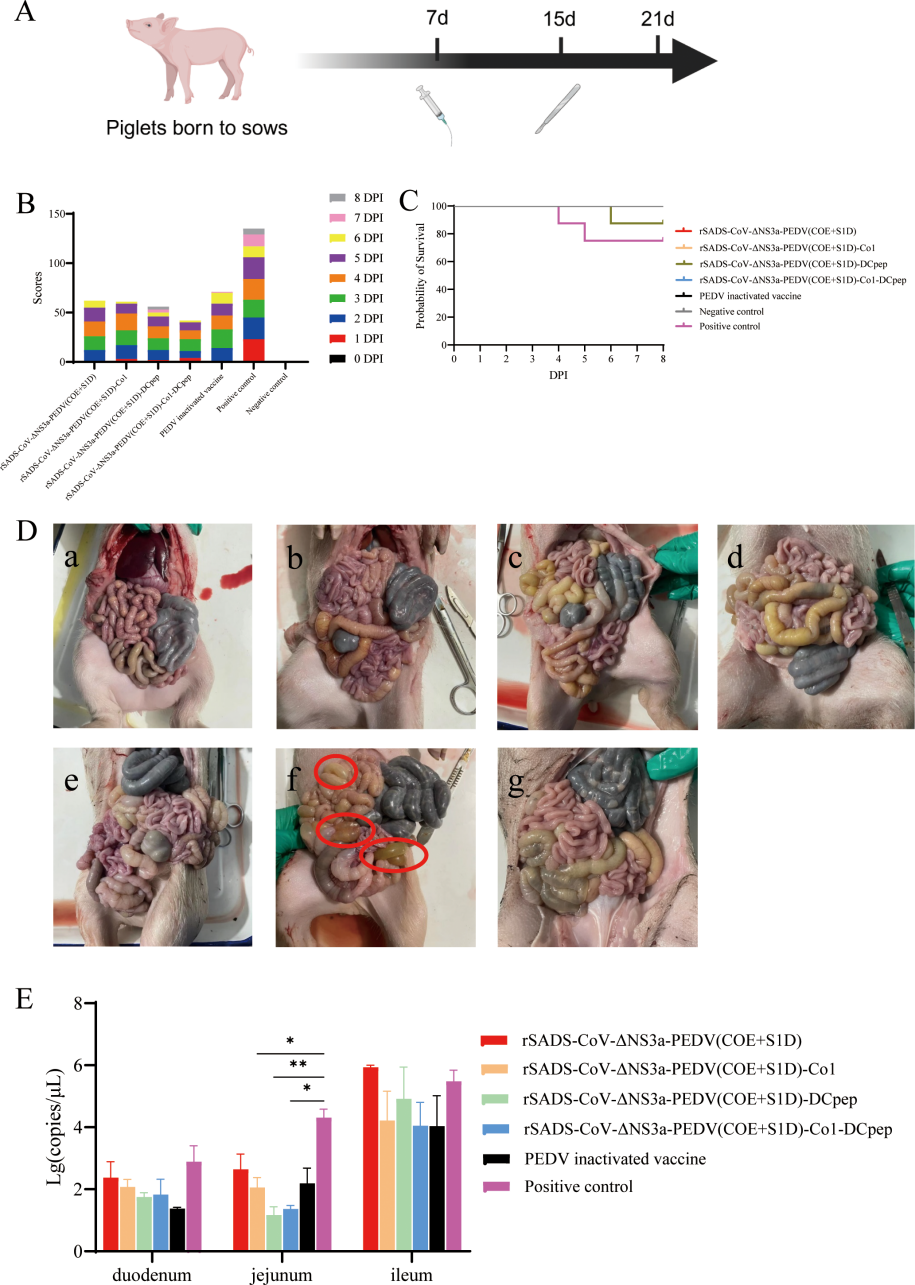
Clinical morbidity and tissue damage in piglets after PEDV challenge. **(A)** Experimental flowchart; piglets born to sows were orally challenged with PEDV-LN at a dose of 10^7.5^ TCID_50_ per piglet, or 10 mL DMEM, and three piglets from each group were randomly selected for necropsy on the eighth day following challenge. **(B)** Cumulative clinical diarrhea score. **(C)** Survival curve of piglets after PEDV challenge. **(D)** Intestinal lesions in piglets after PEDV challenge; a, rSADS-CoV-ΔNS3a-PEDV(COE+S1D) immunogroup; b, rSADS-CoV-ΔNS3a-PEDV(COE+S1D)-Co1 immunogroup; c, rSADS-CoV-ΔNS3a-PEDV(COE+S1D)-DCpep immunogroup; d, rSADS-CoV-ΔNS3a-PEDV(COE+S1D)-Co1-DCpep immunogroup; e, PEDV inactivated vaccine immunogroup; f, positive control (not immunized, PEDV challenge only); g, negative control (not immunized and not challenged with PEDV). The red circles indicate locations where the intestinal wall has thinned significantly. **(E)** Viral copy numbers of PEDV in intestinal tissue after PEDV challenge in piglets. *p < 0.05, **p < 0.01.

To evaluate the clinical protective effects of passive immunization with the recombinant viruses, the piglets were observed daily for clinical symptoms and diarrhea scoring for 8 days post-challenge with PEDV, which was orally administered using 10 mL of 10^6.5^ TCID_50_/mL virus suspension (**Figure 5A**). Results indicated that the negative control group exhibited no illness and accumulated no diarrhea scores, whereas the positive control group displayed severe diarrhea with the highest cumulative diarrhea scores (**Figure 5B**). Diarrhea scores in all recombinant virus-immunized groups were lower than those in the inactivated vaccine group, with the group immunized with the dual-targeting peptide virus rSADS-CoV-ΔNS3a-PEDV(COE+S1D)-Co1-DCpep showing the lowest cumulative scores, indicating superior clinical protection compared to the other three recombinant viruses (**Figure 5B**).

To validate the results on clinical protection of passively immunized piglets, daily survival rates were recorded post-challenge. The survival rate in the positive control group was 75% post-challenge; however, except for one casualty in the rSADS-CoV-ΔNS3a-PEDV(COE+S1D)-DCpep-immunized group due to individual piglet weakness, all other immunized groups maintained a 100% survival rate, demonstrating that immunization of pregnant sows with recombinant strains provided substantial immune protection to their nursing piglets (**Figure 5C**).

To assess the impact of PEDV challenge on piglet weights, daily weigh-ins were conducted for 14 days post-challenge. The positive control piglet group exhibited consistent weight loss within 9 days post-challenge, while piglets in all immunized groups showed a slow increase in weight within the first 7 days, with no significant declines (**Supplementary Figure S4A**). Among the recombinant virus-immunized groups, from day 9 post-challenge onwards, the group immunized with the dual-targeting peptide virus rSADS-CoV-ΔNS3a-PEDV(COE+S1D)-Co1-DCpep experienced the fastest weight gain, indicating better overall condition and suggesting superior clinical protection after immunization with this recombinant virus compared to the other three strains (**Supplementary Figure S4A**).

To evaluate differences in shedding of PEDV among the groups post-challenge, daily rectal swabs were collected for absolute fluorescence quantitative detection of viral RNA (cDNA) copy numbers. Results showed that all groups reached a peak in viral shedding between 3 to 5 days post-challenge, with similar shedding trends post-peak among all immunized groups (**Supplementary Figure S4B**). Compared to the positive control, immunized groups had a shorter duration and lesser amounts of shedding, particularly the rSADS-CoV-ΔNS3a-PEDV(COE+S1D)-Co1-DCpep-immunized group, which had generally lower shedding levels from days 0 to 5 post-challenge, indicating shorter and less impactful effects of PEDV on piglets immunized with this recombinant strain, with quicker recovery observed (**Supplementary Figure S4B**).

On the eighth day following challenge with PEDV, three piglets from each group were randomly selected for necropsy (**Figure 5A**). The piglets in the positive control group exhibited noticeable intestinal wall thinning and transparency, indicating significant pathological changes, whereas no obvious intestinal lesions were observed in piglets from the other groups (**Figure 5D**).

Subsequently, samples of the duodenum, jejunum, and ileum were collected from the necropsied piglets for absolute fluorescence quantitative detection of viral RNA (cDNA) copy numbers for PEDV. The results showed that the PEDV pathogen was present in all sections of the intestine, with the highest viral RNA copy numbers found in the positive control group (**Figure 5E**). Among the immunized groups, the rSADS-CoV-ΔNS3a-PEDV(COE+S1D)-Co1-DCpep group had overall lower viral RNA copy numbers. The copy numbers in the jejunum of piglets immunized with rSADS-CoV-ΔNS3a-PEDV(COE+S1D)-Co1 or rSADS-CoV-ΔNS3a-PEDV(COE+S1D)-Co1-DCpep were significantly lower (P ≤ 0.05) compared to the positive control group, and notably lower (P ≤ 0.01) in the rSADS-CoV-ΔNS3a-PEDV(COE+S1D)-DCpep group. Among the four recombinant virus-immunized groups, the three with genomic inserts for targeting peptides showed lower viral RNA copy numbers in the small intestine tissues compared to the rSADS-CoV-ΔNS3a-PEDV(COE+S1D)-immunized group, indicating that recombinant viruses with targeting peptides can better protect the small intestine tissues of passively immunized piglets from PEDV infection (**Figure 5E**).

**Figure 6:**
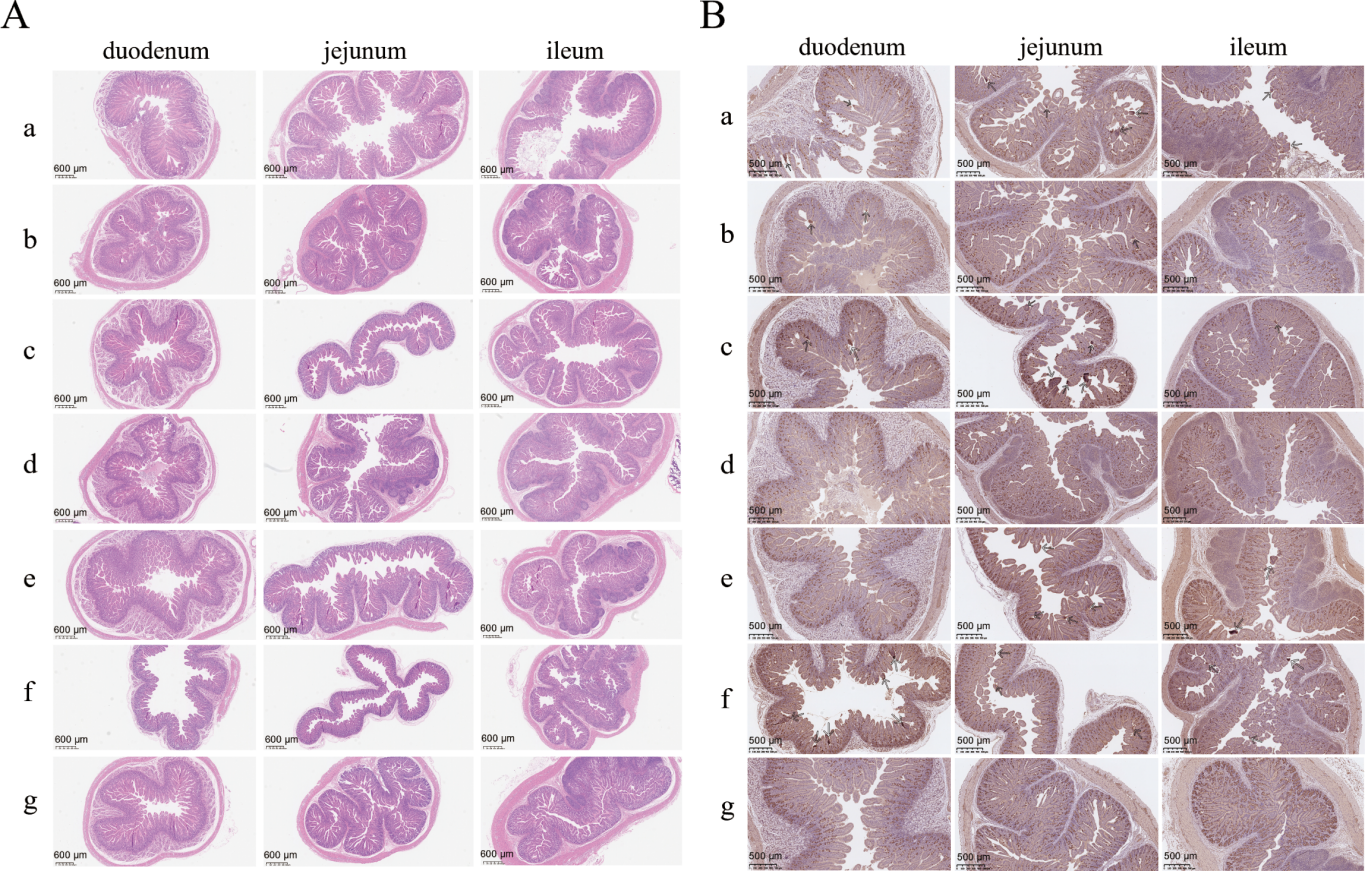
Histopathological analysis of small intestines of piglets after PEDV challenge. **(A)** H&E staining and **(B)** IHC analysis targeting the PEDV spike protein using histopathological sections of piglet small intestines; a, rSADS-CoV-ΔNS3a-PEDV(COE+S1D) immunogroup; b, rSADS-CoV-ΔNS3a-PEDV(COE+S1D)-Co1 immunogroup; c, rSADS-CoV-ΔNS3a-PEDV(COE+S1D)-DCpep immunogroup; d, rSADS-CoV-ΔNS3a-PEDV(COE+S1D)-Co1-DCpep immunogroup; e, PEDV inactivated vaccine immunogroup; f, positive control (not immunized, PEDV challenge only); g, negative control (not immunized and not challenged with PEDV). Arrows indicate the location of intense DAB staining.

Tissue sections of the duodenum, jejunum, and ileum from the piglets post-viral challenge were subjected to hematoxylin and eosin (H&E) staining and immunohistochemistry (**Figure 6**). The piglets in the positive control group showed significant intestinal tissue damage post-PEDV challenge, with poor integrity of the intestinal villi. In contrast, among the other groups, only the piglets in the rSADS-CoV-ΔNS3a-PEDV(COE+S1D)-DCpep-immunized group exhibited severe damage in the jejunum. The intestinal villi of piglets in the other groups were intact, and their intestinal tissues showed no apparent damage (**Figure 6A**). Staining for the PEDV spike protein in the small intestine tissues of the positive control group was pronounced post-PEDV challenge, whereas it was less extensive in the other experimental groups (**Figure 6B**).

## 3 Discussion

Building on previous research suggesting that the accessory proteins of SADS-CoV may influence viral virulence (3), this study demonstrated that the deletion of the SADS-CoV NS3a gene slightly attenuates viral virulence, reducing the damage to intestinal tissues. This modest effect may stem from NS3a’s specialized role in modulating localized inflammatory responses, where its absence imposes limited impact on overall pathogenesis. Moreover, the specific pathways through which NS3a affects virulence were screened and preliminarily validated in our separate recently published study (30). Consequently, we evaluated the potential of SADS-CoV as a vaccine vector by deleting the NS3a site and inserting the sequences for PEDV antigens and for peptides (Co1 and DCpep) that target cells important in the intestinal immune response. Our key findings indicate that the recombinant virus effectively induced cytopathic effects, such as syncytia formation, while maintaining viral genomic stability and replication capacity. These results validate the robustness and efficiency of the SADS-CoV reverse genetics platform, which offers a novel approach for developing vaccines against porcine diseases.

Due to ongoing mutations in circulating strains of PEDV, existing vaccines for PEDV are insufficient to control the spread of the virus (31). Previous researchers have developed viral vector vaccines using various expression systems that focused on the PEDV S (spike) protein, particularly targeting the COE region of the S protein and, to a lesser extent, the entire S1 subunit. However, most vaccines based on single or multiple epitope concatenations have predominantly been subunit vaccines with lower immunogenicity (32). To evaluate the potential of SADS-CoV as a vector for PEDV vaccine development, we selected COE and S1D (aa 499-789) as the protective antigens for PEDV and incorporated targeting peptides for M cells (Co1) and DCs (DCpep) to construct a recombinant SADS-CoV vector expressing the protective PEDV antigens fused with the targeting peptides.

Studies by Huang et al. demonstrated that oral immunization of mice with *Lactobacillus plantarum* vectors expressing the DC-targeting peptide DCpep enhanced the expression of CD11c and CD80 in DCs in fluid of the small intestine when compared to a negative control group (33). Lin et al. constructed a *Bacillus subtilis* vector expressing the PEDV S protein and carrying an M cell targeting peptide; oral immunization of mice with the vector resulted in increased percentages of T cell subgroups CD4^+^ and CD8^+^ in the mesenteric lymph nodes and spleen, and higher IgG levels in mouse serum compared to the control group (34). Consistent with the findings of Huang et al. with the *Lactobacillus plantarum* vectors, our flow cytometric analysis showed that the groups immunized with recombinant viruses containing targeting peptide sequences had higher proportions of CD80 and CD11c cells when compared to the inactivated vaccine group and the non-targeting peptide recombinant virus group, with the rSADS-CoV-ΔNS3a-PEDV(COE+S1D)-Co1-immunized group showing a significant difference in percentage of CD11c cells when compared to the negative control group. The groups immunized with recombinant viruses with the single targeting peptide Co1 and dual targeting peptides Co1-DCpep had higher proportions of CD3^+^CD4^+^ and CD3^+^CD8^+^ cells when compared to the inactivated vaccine group and the non-targeting peptide recombinant virus group. These findings suggest that the significant increase in CD11c^+^/CD80^+^ DCs and CD3^+^CD4^+^/CD8^+^ T cells in sows immunized with Co1-containing viruses directly demonstrates enhanced antigen presentation and Th1/Th2 responses, validating the functionality of the targeting peptides. However, with the recombinant virus with the single targeting peptide DCpep, the ratios of CD3^+^CD4^+^ and CD3^+^CD8^+^ were lower compared to other immunized groups, indicating that this recombinant virus did not effectively stimulate cellular immune responses in sows. This finding is inconsistent with previous research. The inconsistency may stem from inefficient dendritic cell targeting via Houhai acupoint injection or conformational instability of single targeting peptide DCpep, which bypasses natural mucosal routes. By contrast, dual-targeting (Co1-DCpep) leverages M-cell translocation to enhance DCpep delivery, overcoming this limitation. Future work could include structural prediction and mass spectrometry analysis of the antigen proteins to assess stability and receptor binding effects. Additionally, substantial inter-individual variability within the same group was observed, likely attributed to the limited sample size or inherent heterogeneity in immune responses among sows. Larger-scale trials should be conducted to validate the efficacy of the targeting peptides.

Observations on post-challenge pathological changes, including intestinal tissue damage, showed that piglets in the rSADS-CoV-ΔNS3a-PEDV(COE+S1D)-DCpep immunized group had severe damage in the jejunum, while the other groups of piglets had intact small intestinal villi and no obvious intestinal tissue damage. Combined with the finding that the group immunized with the recombinant virus with the single targeting peptide DCpep had lower proportions of CD3^+^CD4^+^ and CD3^+^CD8^+^ cells and therefore could not stimulate better cellular immune responses in sows, we speculate that the occurrence of jejunal damage in this immunized group may be due to insufficient levels of cellular immune responses stimulated by the DC-targeting peptide in sows.

Ma et al. used *Lactococcus lactis* 393 as an antigen carrier for PEDV COE fused with cell-targeting peptides, and their oral immunization experiments in mice showed that the experimental group receiving the cell-targeting peptides had higher IgG and secretory IgA levels than the control group without cell-targeting peptides, indicating better stimulation of immunogenicity (35). Using a DC-targeting vaccine based on PEDV S1 protein, Subramaniam et al. conducted a passive immunization viral challenge protection test, immunizing pregnant sows via Houhai acupoint injections at 5 weeks and 2 weeks before parturition; analysis of PEDV S1-specific antibody IgG and IgA levels in sow peripheral blood and colostrum by ELISA showed detectable IgG antibodies, but no IgA antibodies in serum at any time point (36). Our ELISA testing of PEDV-specific S protein IgG and IgA antibody levels in pre-challenge serum from piglets revealed generally higher serum IgG antibody levels in the immunized groups with targeting peptides, while IgA was almost undetectable, even lower than in the non-targeting peptide recombinant virus-immunized group (This may be attributed to the slightly lower replication rate of the targeting peptide-containing virus observed in the one-step growth curve in porcine cells, the underlying cause of which warrants further investigation). Our findings suggest that the recombinant viruses with targeting peptide sequences that we constructed in this study can stimulate the production of IgG in sows, but cannot effectively stimulate the production of IgA, consistent with the findings of Subramaniam et al. (36). This further suggests that differences in vector and immunization route influence the immunogenicity of vaccines using the same antigen. Future studies should evaluate oral/nasal routes to exploit SADS-CoV’s enteric tropism for IgA induction.

From the cumulative diarrhea scoring of piglets post-challenge, the positive control group showed obvious severe diarrhea and had the highest cumulative diarrhea score; however, the four recombinant virus-immunized groups had lower diarrhea scores than found in the group immunized with the inactivated PEDV vaccine, indicating better clinical protection by the four recombinant viruses. Additionally, the survival curve of piglets post-challenge showed that immunization of pregnant sows with the recombinant strains could provide good immune protection for their nursing piglets. Notably, based on the cumulative daily weight gain of the piglets post-challenge, rSADS-CoV-ΔNS3a-PEDV(COE+S1D)-Co1-DCpep provided better clinical protection compared to the other three recombinant viruses. Similarly, during the peak period of fecal shedding of PEDV, shedding was lower in the rSADS-CoV-ΔNS3a-PEDV(COE+S1D)-Co1-DCpep-immunized group than in the other experimental groups, indicating that, during this period, the impact of PEDV was smaller on piglets immunized with this recombinant strain, and the piglets recovered faster.

Quantitative detection of the PEDV nucleocapsid (N) gene in the intestines of piglets post-challenge showed that, among the four recombinant virus-immunized groups, the three groups with targeting peptides had lower viral RNA copy numbers of PEDV in the small intestinal tissues when compared to the rSADS-CoV-ΔNS3a-PEDV(COE+S1D)-immunized group. Therefore, these findings indicate that immunization of pregnant sows with recombinant viruses expressing these targeting peptides can provide good immune protection for their nursing piglets, reduce clinical symptoms, mitigate the negative impact of PEDV post-challenge on piglets, accelerate recovery post-challenge, reduce organ damage caused by post-challenge viral infections, and suppress PEDV replication in piglet small intestinal tissues, with the rSADS-CoV-ΔNS3a-PEDV(COE+S1D)-Co1-DCpep-immunized group performing the best overall, potentially due to a synergistic effect of the dual-targeting peptides Co1-DCpep. Co1 mediates transepithelial transport to gut-associated lymphoid tissue, while DCpep facilitates dendritic cell uptake, creating an ‘antigen depot’ that amplifies immune priming.

Compared to current inactivated and attenuated live vaccines, SADS-CoV vector vaccines offer advantages in safety, lower production costs, and enhanced stimulation of mucosal immunity. Additionally, this study demonstrated that the combined use of SADS-CoV with mucosal cell-targeting peptides further enhanced the stimulation of mucosal immunity, providing a novel strategy and approach for the development of vaccines against porcine diarrhea. The recombinant viruses constructed in this study have the potential to serve as candidate strains for a new anti-PEDV vaccine based on the SADS-CoV vector, providing more possibilities for further research and development of SADS-CoV vector vaccines, including the development of a multivalent, self-adjuvanted SADS-CoV-based vaccine that co-expresses antigen genes of PEDV, porcine deltacoronaviruses, and rotavirus or other viruses that impact porcine health.

In summary, this study successfully established a strategy for developing a viral vaccine using SADS-CoV as the vector. The constructed recombinant viruses have good proliferation characteristics, stable expression of exogenous proteins, as well as good safety and immunogenicity for pregnant sows. Therefore, our findings indicate that SADS-CoV has the potential to be an excellent vector for the development of vaccines against porcine pathogens.

## 4 Methods

### 4.1 Ethics statement

This study was carried out in accordance with the recommendations of the National Standards for Laboratory Animals of the People’s Republic of China (GB149258-2010). The protocol was approved by Animal Research Committees of South China Agricultural University. Pigs used for the study were handled in accordance with good animal practices required by the Animal Ethics Procedures and Guidelines of the People’s Republic of China.

### 4.2 Cells and viruses

The SADS-CoV/GDWT/2017 strain (GenBank accession no. MG557844), was isolated in Guangzhou, China, in April 2017. The highly pathogenic GII subtype porcine epidemic diarrhea virus (PEDV) strain PEDV-LN was isolated in Guangzhou, China, in 2018.

African green monkey kidney cells (Vero) were cultured in Dulbecco’s modified Eagle’s medium (DMEM) with 10% (vol/vol) fetal bovine serum and 1% (vol/vol) penicillin-streptomycin. Vero cells were used to rescue SADS-CoV/GDWT/2017-derived recombinants, propagate PEDV, and determine the titer of SADS-CoV/GDWT/2017-derived recombinants.

### 4.3 *E. coli* strains and plasmids

*E. coli* GB08-red-gyrA462 was used for deletion of the virulence genes and insertion of genes for antigens. *E. coli* GB2005 was used for transformation with plasmids constructed by Gibson assembly. pBeloBAC11-spect-ccdB-hyg was used to amplify the linear pBeloBAC11-spect vector for construction of the intermediate plasmids pBeloBAC11-spect-CI-SADS-CoV-WT-F1-F6 and pBeloBAC11-spect-SADS-CoV-WT-F7-F10. p15A-amp-ccdB was used to amplify the linear p15A-amp vector for construction of the intermediate plasmid p15A-amp-SADS-CoV-WT-F11-F14-HDVrz-SV40polyA. pBeloBAC11-cm-ccdB-hyg was used to amplify the linear pBeloBAC11-cm vector for construction of the intermediate plasmid pBeloBAC11-cm-CI-SADS-CoV-WT-F1-F14-HDVrz-SV40polyA. pCI-amp-ccdB was used as template to amplify the human cytomegalovirus (CMV) immediate-early promoter. R6K-mCMV-GFP-SV40pA-FRT-amp was used as template to amplify SV40polyA. pCI-neo (Promega) was used to amplify the linear pCI-amp vector for construction of the helper plasmid pCI-amp-SADS-CoV-N. p15A-amp-ccdB was used as template to amplify the amp-ccdB resistance gene. The plasmids and bacterial strains used in this study can be freely requested from our laboratory (Shandong University) for academic research.

### 4.4 Construction of SADS-CoV infectious clones

SADS-CoV genomic RNA was extracted from Vero cells infected with SADS-CoV/GDWT/2017 using an AxyPrep Body Fluid Viral DNA/RNA Miniprep Kit (Axygen) according to the manufacturer’s instructions. The extracted RNA was then reverse transcribed using the PrimeScript™ 1st Strand cDNA Synthesis Kit (Takara Bio) according to the manufacturer’s instructions. Fourteen fragments covering the SADS-CoV genome, labeled F1 through F14, were obtained through PCR amplification (**Supplementary Table S1**). The fragments pBeloBAC11-cm, pBeloBAC11-spect(F1-F6), pBeloBAC11-spect(F7-F10), p15A-amp(F11-F4), CMV, and SV40polyA were obtained through PCR amplification (**Supplementary Table S1**). After gel electrophoresis, the linear fragments were recovered from the gel and set aside for further use. Additionally, the HDVrz (ribozyme) fragment was synthesized through full gene synthesis and prepared as a water-soluble dry powder for future use.

The pBeloBAC11-spect-CI-SADS-CoV-WT-F1-F6, pBeloBAC11-spect-SADS-CoV-WT-F7-F10, and p15A-amp-SADS-CoV-WT-F11-F14-HDVrz-SV40polyA plasmids were constructed by Gibson assembly using the Gibson Assembly Cloning Kit (NEB). The optimal ratio of the amounts of genomic and vector DNA was determined by titration. After dialyzing, the Gibson-assembled DNA mixture was electroporated into *E. coli* GB2005. The intermediate plasmids pBeloBAC11-spect-CI-SADS-CoV-WT-F1-F6, pBeloBAC11-spect-SADS-CoV-WT-F7-F10, and p15A-amp-SADS-CoV-WT-F11-F14-HDVrz-SV40polyA were selected on LB plates containing 60 μg/ml spectinomycin or 100 μg/ml ampicillin, and the correct recombinants were identified by PvuII, PstI, or EcoRV restriction enzyme analysis, respectively. The linear fragments CI-F1-F6, F7-F10, and F11-F14-HDVrz-SV40polyA were obtained by removing the vector portion of each intermediate plasmid through digestion at the pre-set NotI+XbaI/EcoRV/EcoRV restriction sites at both ends of the vector. The aforementioned fragments were combined with the PCR-amplified fragment pBeloBAC11-cm using Gibson assembly and transformed into *E. coli* GB2005 competent cells to obtain the full-length recombinant vector pBeloBAC11-cm-CI-SADS-CoV-WT-F1-F14-HDVrz-SV40polyA. The recombinants were selected on LB plates containing 15 μg/ml chloramphenicol. The correct recombinants were identified by XmnI restriction enzyme analysis. Sequencing of these plasmids was conducted by Sangon.

The fragments pCI-amp and SADS-CoV-N were obtained through PCR amplification (**Supplementary Table S1**), and the helper plasmid pCI-amp-SADS-CoV-N was then constructed by Gibson assembly and transformed into *E. coli* GB2005 competent cells. The recombinants were selected on LB plates containing 100 μg/ml ampicillin, and the correct recombinants were identified by BanI restriction enzyme analysis. Sequencing of the helper plasmid was conducted by Sangon.

The infectious clone and the helper plasmid pCI-amp-SADS-CoV-N were transfected into Vero cells using Lipofectamine 3000 (Thermo Fisher Scientific) to rescue the virus rSADS-CoV.

### 4.5 Knockout of the accessory protein NS3a gene

The fragment ΔNS3a-amp-ccdB(knockout) was obtained through PCR amplification, and the homologous arms with AscI restriction sites were incorporated in the oligonucleotides (**Supplementary Table S1**). After gel electrophoresis, the linear fragment was recovered from the gel and set aside for further use. Using the infectious clone pBeloBAC11-cm-CI-SADS-CoV-WT-F1-F14-HDVrz-SV40polyA, the NS3a gene was deleted by Redαβ recombineering in *E. coli* GB08-red-gyrA462. The recombinants were selected on LB plates containing 100 μg/ml ampicillin and 15 μg/ml chloramphenicol. After confirmation by restriction enzyme analysis, the amp-ccdB segment of the correct clones was removed by AscI digestion. The resulting linear fragment was then combined using Gibson assembly and transformed into *E. coli* GB2005 to obtain the recombinant plasmid pBeloBAC11-cm-CI-SADS-CoV-WT-F1-F14-HDVrz-SV40polyA-ΔNS3a. The recombinants were selected on LB plates containing 15 μg/ml chloramphenicol, and the correct recombinants were identified by PstI restriction enzyme analysis and PCR analysis (**Supplementary Table S2**). Sequencing of this plasmid was conducted by Sangon.

The infectious clone and the helper plasmid pCI-amp-SADS-CoV-N were transfected into Vero cells using Lipofectamine 3000 to rescue the deletion mutant virus rSADS-CoV-ΔNS3a.

### 4.6 Knockin of the PEDV antigen

PEDV genomic RNA was extracted from Vero cells infected with PEDV using an AxyPrep Body Fluid Viral DNA/RNA Miniprep Kit according to the manufacturer’s instructions. The fragments ΔNS3a-amp-ccdB(knockin) with AscI restriction site, ΔNS3a-PEDV(COE+S1D), ΔNS3a-PEDV(COE+S1D)-Co1, ΔNS3a-PEDV(COE+S1D)-DCpep, and ΔNS3a-PEDV(COE+S1D)-Co1-DCpep were obtained through PCR amplification, and the homologous arms were incorporated in the oligonucleotides (**Supplementary Table S1**). After gel electrophoresis, the linear fragments were recovered from the gel and set aside for further use. Using the infectious clone pBeloBAC11-cm-CI-SADS-CoV-WT-F1-F14-HDVrz-SV40polyA, the NS3a gene was deleted by Redαβ recombineering in *E. coli* GB08-red-gyrA462. The recombinants were selected on LB plates containing 100 μg/ml ampicillin and 15 μg/ml chloramphenicol. After confirmation by restriction enzyme analysis, the amp-ccdB segment of the correct clones was removed by AscI digestion. The resulting linear fragment was then combined with linear fragment ΔNS3a-PEDV(COE+S1D), ΔNS3a-PEDV(COE+S1D)-Co1, ΔNS3a-PEDV(COE+S1D)-DCpep, or ΔNS3a-PEDV(COE+S1D)-Co1-DCpep using Gibson assembly and transformed into *E. coli* GB2005 to obtain the recombinant plasmids pBeloBAC11-SADS-CoV-ΔNS3a-PEDV(COE+S1D), pBeloBAC11-SADS-CoV-ΔNS3a-PEDV(COE+S1D)-Co1, pBeloBAC11-SADS-CoV-ΔNS3a-PEDV(COE+S1D)-DCpep, and pBeloBAC11-SADS-CoV-ΔNS3a-PEDV(COE+S1D)-Co1-DCpep, respectively. The recombinants were selected on LB plates containing 15 μg/ml chloramphenicol, and the correct recombinants were identified by PstI restriction enzyme analysis and PCR analysis (**Supplementary Table S2**). Sequencing of these plasmids was conducted by Sangon.

The infectious clones and the helper plasmid pCI-amp-SADS-CoV-N were transfected into Vero cells using Lipofectamine 3000 to rescue the viruses rSADS-CoV-ΔNS3a-PEDV(COE+S1D), rSADS-CoV-ΔNS3a-PEDV(COE+S1D)-Co1, rSADS-CoV-ΔNS3a-PEDV(COE+S1D)-DCpep, and rSADS-CoV-ΔNS3a-PEDV(COE+S1D)-Co1-DCpep.

### 4.7 Stability of passage of recombinant viruses

The recombinant viruses were inoculated into Vero cells and serially passaged up to the 20th generation, with continuous monitoring of cytopathic effects. RT-PCR was performed on the 2, 5, 8, 11, 14, 17, and 20 passages to confirm the stable presence of the exogenous NS3a gene. A 200 µL sample from the supernatant of each passage of recombinant virus and the positive control rSADS-CoV virus was taken for RNA extraction using a viral DNA/RNA extraction kit (Guangzhou Yueyang Biological Technology Co., Ltd.). The extracted RNA served as a template for amplifying the NS3a gene site using primers SADS-A25-F and SADS-A25-R (**Supplementary Table S2**) with the HiScript II One Step RT-PCR Kit (Vazyme). The RT-PCR products were analyzed by 1% agarose gel electrophoresis to confirm that the product sizes were correct. Sequencing of these products was conducted by Sangon.

### 4.8 One-step growth curve of recombinant viruses

The rSADS-CoV-ΔNS3a-PEDV(COE+S1D) passage 2, rSADS-CoV-ΔNS3a-PEDV(COE+S1D)-Co1 passage 2, rSADS-CoV-ΔNS3a-PEDV(COE+S1D)-DCpep passage 2, and rSADS-CoV-ΔNS3a-PEDV(COE+S1D)-Co1-DCpep passage 2 viruses, along with the parental virus rSADS-CoV, were inoculated into Vero cells and IPI-2I cells at an MOI of 0.01. Cell supernatants were harvested at 0 h, 3 h, 6 h, 9 h, 12 h, 18 h, 24 h, 30 h, 36 h, 48 h, 60 h, and 72 h, and stored at -80°C. Viral RNA was extracted from a 200 µL sample for each time point using the viral DNA/RNA extraction kit (Guangzhou Yueyang Biological Technology Co., Ltd.). Viral RNA served as a template for quantitative real-time PCR using primers SADS-qF, SADS-qR, and the SADS-probe (**Supplementary Table S3**) with the HiScript II U+ One Step qRT-PCR Probe Kit to measure viral copy numbers at each time point.

### 4.9 Western blot identification of exogenous protein expressed by recombinant viruses

Recombinant viruses were inoculated into Vero cells in six-well plates and incubated at 37°C for 2 h, followed by replacement of the medium with DMEM containing 8 µg/mL trypsin and further incubation at 37°C, 5% CO_2_. After 24 h, cells were washed three times with PBS and lysed in 1% protease inhibitor-containing RIPA Lysis Buffer (CWBIO) at 4°C for 30 min. The lysates were collected in 1.5 mL centrifuge tubes, mixed with a quarter volume of 5×SDS loading buffer (CWBIO), and boiled for 10 min to extract total protein. Proteins were separated on a 12.5% PAGE gel using the PAGE Gel Fast Preparation kit (Epizyme) for separation and stacking gels, with electrophoresis at 100 V. Proteins were then transferred to a PVDF membrane using a semi-dry transfer method. The membrane was blocked at room temperature in 5% skim milk in TBST for 2 h, incubated overnight at 4°C with PEDV S mouse pAb diluted 1:1000 in TBST, washed five times with TBST, and then incubated with Multi-rAb HRP-Goat Anti-Mouse Recombinant Secondary Antibody (H+L) (Proteintech, RGAM001), diluted 1:10000 in TBST, at room temperature for 1 h. After washing, the membrane was developed using an Azure 500 imaging system with the Omni-ECL^™^ Femto Light Chemiluminescence Kit (Epizyme).

### 4.10 Immunofluorescence assay of exogenous protein expressed by recombinant viruses

Recombinant viruses were inoculated into Vero cells in 12-well plates and incubated at 37°C for 2 h with DMEM containing 8 µg/mL trypsin. Once cell cytopathy reached 50%, cells were fixed with 4% paraformaldehyde precooled at 4°C for 30 min, and then the cells were permeabilized with 0.1% Triton-X-100 for 15 min at room temperature. After blocking with 5% skim milk in TBST for 1 h, the cells were incubated overnight at 4°C with PEDV S mouse pAb diluted 1:500 in TBST. After washing, cells were incubated with CoraLite488-conjugated Goat Anti-Mouse IgG(H+L) (Proteintech, SA00013-1) diluted 1:500 in TBST for 1 h, washed again, and then stained with 4’,6-diamidino-2-phenylindole (DAPI). Fluorescence microscopy was used to observe and photograph the expression of green and blue fluorescence indicating the presence of exogenous proteins.

### 4.11 Animal experiments

Twenty-six 5-day-old piglets, which tested negative for SADS-CoV antibodies, antigens, and other diarrheal pathogen antigens, were selected. Of these, 7 piglets were orally administered 10 mL of 10^-6.8^ TCID50/mL rSADS virus suspension, 7 piglets were orally administered 10 mL of 10^-6.8^ TCID50/mL rSADS-ΔNS3a virus suspension, and the remaining 5 piglets served as negative controls and were orally administered 10 mL of DMEM. Observations continued for 14 days post-challenge.

Thirty-six pregnant sows, negative for PEDV antibodies and antigens, were selected and divided into six groups of six each. Recombinant viruses rSADS-CoV-ΔNS3a-PEDV(COE+S1D), rSADS-CoV-ΔNS3a-PEDV(COE+S1D)-Co1, rSADS-CoV-ΔNS3a-PEDV(COE+S1D)-DCpep, and rSADS-CoV-ΔNS3a-PEDV(COE+S1D)-Co1-DCpep, along with a commercial inactivated PEDV vaccine and DMEM, were administered via Houhai acupoint injections to the sows 30 days before parturition, with a booster given 9 days before delivery. The negative control group received DMEM. Immunization grouping and doses are detailed in **Table 1**. Piglets aged 7 days from groups M1 to M5 and 16 piglets from group M6 (including negative and positive control groups) were orally challenged with PEDV-LN at a dose of 10^7.5^ TCID_50_ per piglet; the negative control group received 10 mL of DMEM per piglet. Observations continued for 14 days post-challenge.

Following the SADS-CoV/PEDV challenge, clinical symptoms and diarrhea scores were observed daily. Clinical symptom scoring criteria for diarrhea were: 0 points for no diarrhea, 1 point for pasty feces, 2 points for loose stool, and 3 points for watery diarrhea/death. Piglets were weighed daily, and cumulative daily weight gain was counted.

### 4.12 Detection of DC subsets and T lymphocyte subsets by flow cytometry

At parturition, peripheral blood was collected from the sows, and flow cytometry was used to measure DC subgroups CD11c and CD80, and T lymphocyte subgroups CD3^+^CD4^+^ and CD3^+^CD8^+^. The specific detection steps were as follows: each sample was divided into three groups, with 200 μL of whole blood used for detection in each group. The first group received 10 μL of Mouse Anti-Porcine CD3ϵ-SPRD (PPT3) (SouthernBiotech), 2 μL of Mouse Anti-Porcine CD4-APC (74-12-4) (SouthernBiotech, 4515-11), and 10 μL of Mouse Anti-Porcine CD8α-AF488 (76-2-11) (SouthernBiotech, 4520-30). The second group received 1.5 μL of CD11c/Integrin Alpha X polyclonal antibody (Proteintech, 17342-1-AP), and the third group received 1.5 μL of CD80/B7-1 monoclonal antibody (Proteintech, 66406-1-Ig). The samples were mixed thoroughly and incubated in the dark at room temperature for 20 minutes. Subsequently, 2 mL of 1× hemolysin was added, followed by vortexing and incubation in the dark at room temperature for 20 minutes. The samples were then centrifuged at 1000 r/min for 10 minutes, the supernatant was discarded, and the cells were washed twice with PBS. The cells in the first group were resuspended in 500 μL of PBS for analysis. The second group received 2 μL of FITC-conjugated AffiniPure Goat Anti-rabbit IgG (H+L) (Boster Bio, BA1105), and the third group received 2 μL of Goat Anti-mouse IgG/Alexa Fluor 488 (Bioss, bs-0296G-AF488); samples were then incubated in the dark at 4°C for 30 minutes, followed by two PBS washes, and resuspension in 500 μL of PBS for analysis. The resuspended cells from all three groups were then subjected to flow cytometry analysis using a BD FACSCalibur.

### 4.13 ELISA assays of PEDV IgG and IgA

Serum samples were collected from sows in labor and piglets before viral challenge to assess PEDV-specific S protein IgG and IgA levels using the PEDV IgG ELISA kit and PEDV IgA ELISA kit, both obtained from the Guangzhou Yueyang Biological Technology Co., Ltd.

### 4.14 Detection of viral copy number in anal swabs of piglets after challenge

Daily post-challenge, rectal swabs were collected and immersed in centrifuge tubes containing 1 mL of PBS. After vortexing, the swab samples were centrifuged at 10,000 rpm at room temperature for 1 minute. A 200 µL aliquot of the supernatant was used to extract viral RNA using a viral DNA/RNA extraction kit (Guangzhou Yueyang Biological Technology Co., Ltd.). Viral RNA of SADS-CoV/PEDV served as a template for quantitative real-time PCR (**Supplementary Table S3**), using the protocol for the HiScript II U+ One Step qRT-PCR Probe Kit.

### 4.15 Small intestinal tissue sample collection

Due to the requirements of tissue sample collection in this experiment, piglets were euthanized using the exsanguination method following anesthesia to minimize the effect of blood on the tissue samples. Zoletil^®^50 (Virbac) was diluted according to the manufacturer’s instructions and administered intramuscularly at a dose of 20 mg/kg body weight. After the piglets became unconscious, with relaxed muscles, stable breathing, and the absence of corneal reflex, the carotid artery was severed using a scalpel to induce exsanguination and euthanasia. Four days after SADS-CoV challenge and eight days after PEDV challenge, three piglets from each group were randomly selected for necropsy after euthanasia by the method described above to document and photograph intestinal lesions. Samples from the duodenum, jejunum, and ileum were collected. Each tissue sample, weighing 0.5 g, was placed in a grinding tube with 1 mL PBS and grinding beads, and homogenized. After centrifugation at 10,000 rpm for 2 minutes, 200 µL of the supernatant was extracted using the viral DNA/RNA extraction kit. Viral RNA of SADS-CoV and PEDV served as a template for quantitative real-time PCR (**Supplementary Table S3**), following the protocol for the HiScript II U+ One Step qRT-PCR Probe Kit to determine viral copy numbers.

### 4.16 Hematoxylin and eosin staining for intestinal histopathology

Samples of 1-2 cm in size from the duodenum, jejunum, and ileum of each group were fixed in 4% formaldehyde solution for 48 hours, and then embedded in paraffin, sectioned, and subjected to H&E staining. Dehydration was performed stepwise using low to high concentrations of alcohol, followed by embedding in paraffin and further processing and then preparation of tissue sections using standard procedures. The sections were stained with Harris hematoxylin for 3-5 minutes, rinsed in tap water for 1-2 minutes, differentiated in 0.8%-1% hydrochloric acid alcohol, and rinsed again in tap water. Bluing was performed in a diluted lithium carbonate solution, followed by rinsing for 1-2 minutes, and then staining in eosin (alcohol-soluble) for 1-2 seconds. Dehydration was performed by immersing the sections in 95% ethanol and absolute ethanol for 1-2 minutes each. The sections were then cleared in xylene, mounted, and scanned with a brightfield scanner.

### 4.17 Immunohistochemistry of intestinal tissue

Separate sections of the above paraffin-embedded samples were processed in synchrony for IHC. Paraffin-embedded sections were deparaffinized three times with an eco-friendly deparaffinizing agent for 10 minutes each and then rehydrated through a graded series of ethanol: absolute, 95%, and 75% for 5 minutes each. Samples were washed three times with Tris-buffered saline (TBS), 3 minutes per wash, and then citrate buffer (pH 6.0) was added prior to further processing by boiling, cooling, and then washing three times with TBS, 5 minutes per wash, before incubation in 3% hydrogen peroxide at room temperature for 30 minutes to block endogenous peroxidase activity. Samples were then rinsed three times with TBS, 5 minutes per wash, placed in TBST, and then blocked with 10% goat serum at room temperature for 30 minutes. After discarding the serum, approximately 50 µl per section (dependent on tissue size) of primary antibody (anti-PEDV S protein mouse polyclonal antibody), which was diluted 1:1000 in 10% goat serum, was added followed by incubation overnight at 4°C. After washing three times with TBST, goat anti-mouse secondary antibody was added followed by incubation at 37°C for 45 minutes, and washing again three times with TBST. After discarding the TBST, 50 µl of freshly prepared DAB was added per section (dependent on tissue size), and the reaction was terminated with tap water after an appropriate period of time. Samples were then processed by staining with hematoxylin and bluing solution to colour the nuclei and then mounting using standard protocols prior to scanning with a brightfield scanner.

### 4.18 Data analysis

Differences between groups were analyzed using GraphPad Prism 8.0 software. Experimental data were presented as mean ± standard error of the mean (SEM). A *p*-value > 0.05 was considered “not significant”, *p* ≤ 0.05 was considered “significant” (denoted by *), and *p* ≤ 0.01 was considered “highly significant” (denoted by **).

## Data Availability

The original contributions presented in the study are included in the article/**Supplementary Material**. Further inquiries can be directed to the corresponding authors.
