# Supplementary material for: Recombinant SADS-CoV as a vector for porcine epidemic diarrhea vaccine development
Source: Front Immunol. 2025 Aug 6;16:1633661. doi: 10.3389/fimmu.2025.1633661 (PMC12364652; doi:10.3389/fimmu.2025.1633661)
Supplement: Supplementary file 1 [file Table1.docx]

***
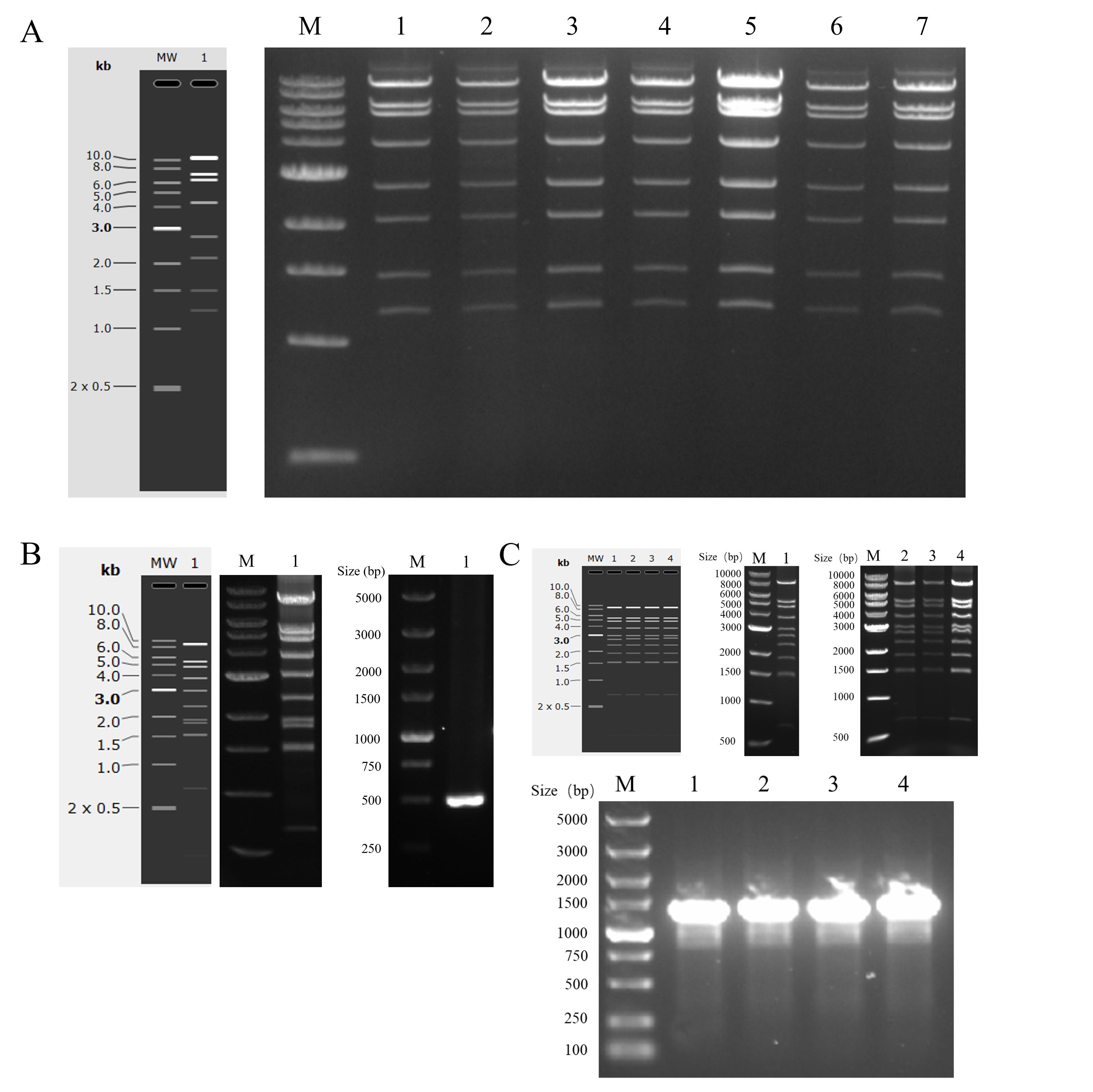
***

**Fig. S1. PCR and restriction enzyme digest confirmation of the SADS-CoV full-length infectious clone and the recombinant viral plasmids.** (A) Confirmation of the SADS-CoV full-length infectious clone by XmnI digest. (B) PCR and enzymatic confirmation of recombinant viral plasmid lacking NS3a. (C) PCR and enzymatic confirmation of recombinant viral plasmids with PEDV antigen sequences; lanes 1-4 show exogenous gene inserts in pBeloBAC11-SADS-CoV-ΔNS3a-PEDV(COE+S1D), pBeloBAC11-SADS-CoV-ΔNS3a-PEDV(COE+S1D)-Co1, pBeloBAC11-SADS-CoV-ΔNS3a-PEDV(COE+S1D)-DCpep, and pBeloBAC11-SADS-CoV-ΔNS3a-PEDV(COE+S1D)-Co1-DCpep, respectively.

***
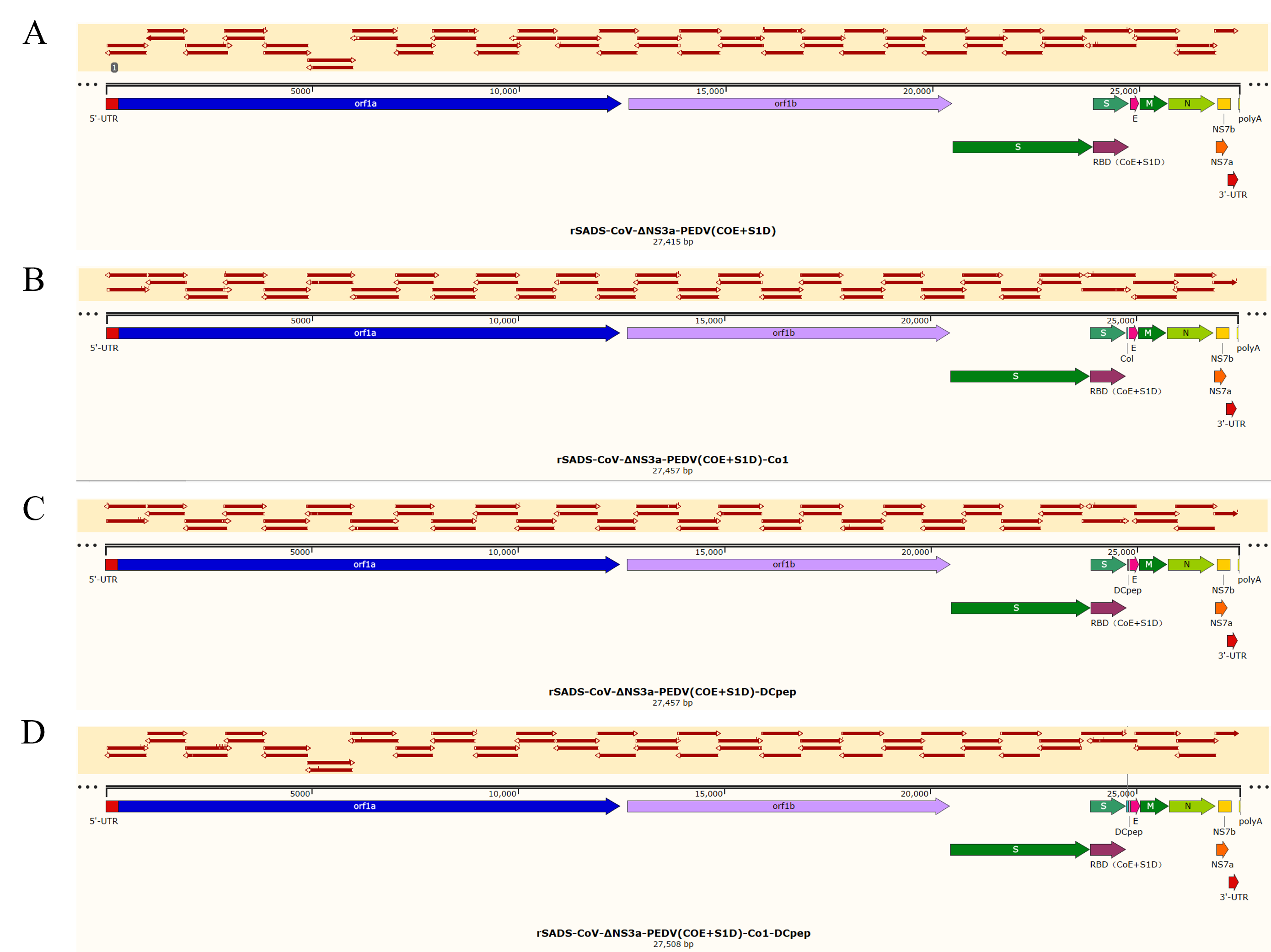
***

**Fig. S2.** **PCR-based assessment of full-length genomic stability in recombinant viruses after 20 passages.**(A-D) Sequence alignment results of: (A) rSADS-CoV-ΔNS3a-PEDV(COE+S1D), (B) rSADS-CoV-ΔNS3a-PEDV(COE+S1D)-Co1, (C) rSADS-CoV-ΔNS3a-PEDV(COE+S1D)-DCpep, and (D) rSADS-CoV-ΔNS3a-PEDV(COE+S1D)-Co1-DCpep.

******

******

**Fig. S3. Flow cytometric analysis of dendritic cell subsets CD11c/CD80 and T cell subsets CD3/CD3^+^CD4^+^/CD3^+^CD8^+^ in peripheral blood of sows.** (A-F) CD11c cells in the (A) rSADS-CoV-ΔNS3a-PEDV(COE+S1D), (B) rSADS-CoV-ΔNS3a-PEDV(COE+S1D)-Co1, (C) rSADS-CoV-ΔNS3a-PEDV(COE+S1D)-DCpep, and (D) rSADS-CoV-ΔNS3a-PEDV(COE+S1D)-Co1-DCpep immunized groups, (E) PEDV inactivated vaccine immunized group, and (F) mock group. (G-L) CD80 cells in the (G) rSADS-CoV-ΔNS3a-PEDV(COE+S1D), (H) rSADS-CoV-ΔNS3a-PEDV(COE+S1D)-Co1, (I) rSADS-CoV-ΔNS3a-PEDV(COE+S1D)-DCpep, and (J) rSADS-CoV-ΔNS3a-PEDV(COE+S1D)-Co1-DCpep immunized groups, (K) PEDV inactivated vaccine immunized group, and (L) mock group. (M-R) CD3/CD3^+^CD4^+^/CD3^+^CD8^+^ cells in the (M) rSADS-CoV-ΔNS3a-PEDV(COE+S1D), (N) rSADS-CoV-ΔNS3a-PEDV(COE+S1D)-Co1, (O) rSADS-CoV-ΔNS3a-PEDV(COE+S1D)-DCpep, and (P) rSADS-CoV-ΔNS3a-PEDV(COE+S1D)-Co1-DCpep immunized groups, (Q) PEDV inactivated vaccine immunized group, and (R) mock group.

***
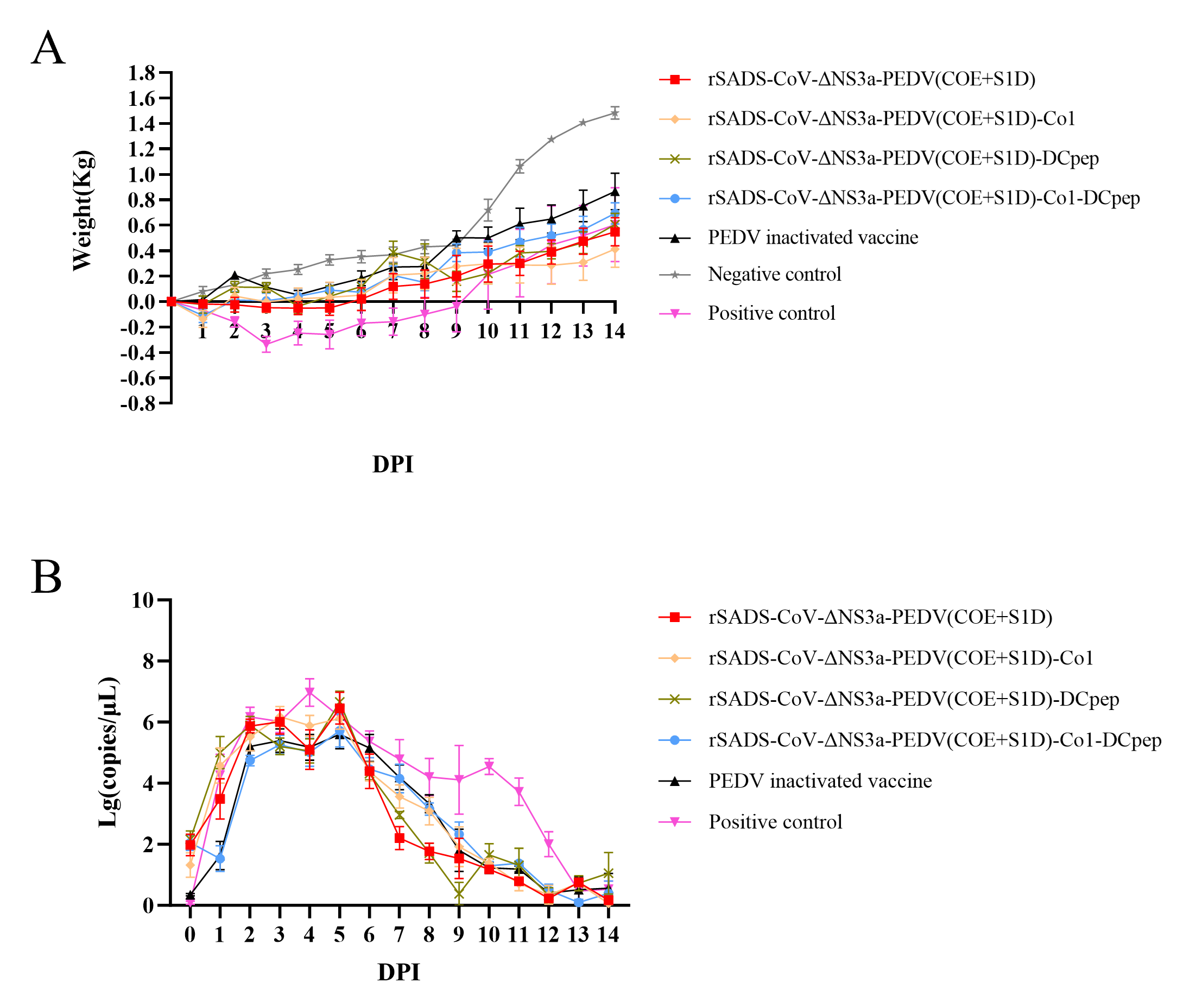
***

**Fig. S4. Cumulative daily weight gain and viral load in anal swabs of piglets after PEDV challenge.** (A) Cumulative daily weight gain. (B) Viral load of PEDV. DPI, days post-infection.

**Table S1.** Primer sequences for amplification of fragments required for construction of SADS-CoV full-length infectious clone and recombinant viral plasmids.

| Primer name | Primer sequence (5’-3’) | Description |
| --- | --- | --- |
| **pBeloBAC11-spect-CI-SADS-CoV-WT-F1-F6** | | |
| BAC-SPEC-1-6-F | TACATAGGTGCTACTGTTAGGTTGCAGGCTGGTAAACAGAGGATCCCTCTAGAGTCGACCTGCAGG | Linear fragment pBeloBAC11-spect for the construction of pBeloBAC11-spect-CI-SADS-CoV-WT-F1-F6 |
| BAC-SPEC-1-6-R | CTATATAACCAATGAATAATATGGCTAATGGCCAATATTGAGCGGCCGCGGGCCCCGGGTACCGAGCTCGAATTC |  |
| SADS-CMV+Intron-F | TCAATATTGGCCATTAGCCATATT | Linear fragment CMV+Intron for the construction of pBeloBAC11-spect-CI-SADS-CoV-WT-F1-F6 |
| SADS-CMV+Intron-R | ACTCTATCGGCAGATAGATTATATCTTTAAGTCCCCATGTCCTATAGTGAGTCGTATTAAGT |  |
| SADS-CoV-F1-F | ACATGGGGACTTAAAGATATAATC | Linear fragment 1 of SADS-CoV genome for the construction of pBeloBAC11-spect-CI-SADS-CoV-WT-F1-F6 |
| SADS-CoV-F1-R | AACATCAGCATCAGCCATAAAC |  |
| SADS-CoV-F2-F | GGTCTGATGGCTATTATAGG | Linear fragment 2 of SADS-CoV genome for the construction of pBeloBAC11-spect-CI-SADS-CoV-WT-F1-F6 |
| SADS-CoV-F2-R | TTAATATCGGCGTCCTTAAC |  |
| SADS-CoV-F3-F | AACACCATTGACACAATAGTTG | Linear fragment 3 of SADS-CoV genome for the construction of pBeloBAC11-spect-CI-SADS-CoV-WT-F1-F6 |
| SADS-CoV-F3-R | GCGTAACTACCATTACGGATAAC |  |
| SADS-CoV-F4-F | CTTGTGGCTTCCTCAGTTGT | Linear fragment 4 of SADS-CoV genome for the construction of pBeloBAC11-spect-CI-SADS-CoV-WT-F1-F6 |
| SADS-CoV-F4-R | TGAGCATGCCAGTCCATAAATG |  |
| SADS-CoV-F5-F | GTTCGCAACACGTTTGACAC | Linear fragment 5 of SADS-CoV genome for the construction of pBeloBAC11-spect-CI-SADS-CoV-WT-F1-F6 |
| SADS-CoV-F5-R | GTGCTCTGTATGTTAACACC |  |
| SADS-CoV-F6-F | GAGGTTGTTAGGCAAATGTATG | Linear fragment 6 of SADS-CoV genome for the construction of pBeloBAC11-spect-CI-SADS-CoV-WT-F1-F6 |
| SADS-CoV-F6-R | TCTGTTTACCAGCCTGCAACC |  |
| **pBeloBAC11-spect-SADS- CoV-WT-F7-F10** | | |
| BAC-SPEC-7-10-F | ATTAATGGATTTATACGTGAAAAACTTGCGCTTGGTGGCGCGATATCCTCTAGAGTCGACCTGCAGG | Linear fragment pBeloBAC11-spect for the construction of pBeloBAC11-spect-SADS- CoV-WT-F7-F10 |
| BAC-SPEC-7-10-R | CTGTTTACCAGCCTGCAACCTAACAGTAGCACCTATGTAGGATATCCGGGTACCGAGCTCGAATTC |  |
| SADS-CoV-F7-F | CTACATAGGTGCTACTGTTAG | Linear fragment 7 of SADS-CoV genome for the construction of pBeloBAC11-spect-SADS- CoV-WT-F7-F10 |
| SADS-CoV-F7-R | AGTTGGTAAGATATTACGCTTTG |  |
| SADS-CoV-F8-F | ACGCACTTTACGCTATGACAAAG | Linear fragment 8 of SADS-CoV genome for the construction of pBeloBAC11-spect-SADS- CoV-WT-F7-F10 |
| SADS-CoV-F8-R | TAGACCAATAACACAATGTG |  |
| SADS-CoV-F9-F | GACCTCCTGGTAGTGGCAAATC | Linear fragment 9 of SADS-CoV genome for the construction of pBeloBAC11-spect-SADS- CoV-WT-F7-F10 |
| SADS-CoV-F9-R | GAATTAATAGGCTGTTTGTC |  |
| SADS-CoV-F10-F | AATGTACCTTGGTACTGTTAC | Linear fragment 10 of SADS-CoV genome for the construction of pBeloBAC11-spect-SADS- CoV-WT-F7-F10 |
| SADS-CoV-F10-R | GCGCCACCAAGCGCAAGTTTTTC |  |
| **p15A-amp-SADS-CoV-WT-F11-F14-HDVrz-SV40polyA** | | |
| P15A-amp-11-14-F | GAGGTTTTTTAAAGCAAGTAAAACCTCTACAAATGTGGTAGATATCGCGGCCGCGCGCTAGCGGAGTGTATACT | Linear fragment p15A-amp for the construction of p15A-amp-SADS-CoV-WT-F11-F14-HDVrz-SV40polyA |
| P15A-amp-11-14-R | CGCCACCAAGCGCAAGTTTTTCACGTATAAATCCATTAATGATATCGCGGCCGCTTACCAATGCTTAATCAGTGA |  |
| SADS-CoV-SV40 polyA-F | GACGTCGTCCACTCGGATGGCTAAGGGAGAGCTCGGATCCAGACATGATAAGATACATTG | Linear fragment SV40 polyA for the construction of p15A-amp-SADS-CoV-WT-F11-F14-HDVrz-SV40polyA |
| SADS-CoV-SV40 polyA-R | TACCACATTTGTAGAGGTTTTAC |  |
| SADS-CoV-F11-F | ATTAATGGATTTATACGTG | Linear fragment 11 of SADS-CoV genome for the construction of p15A-amp-SADS-CoV-WT-F11-F14-HDVrz-SV40polyA |
| SADS-CoV-F11-R | ATTTACCATGGTGTCATTAG |  |
| SADS-CoV-F12-F | TGTTTAATGCTAGTTACGCAC | Linear fragment 12 of SADS-CoV genome for the construction of p15A-amp-SADS-CoV-WT-F11-F14-HDVrz-SV40polyA |
| SADS-CoV-F12-R | GCAAAGTACCACACAAACATG |  |
| SADS-CoV-F13-F | CTGGGCTATATGTTGACTAGC | Linear fragment 13 of SADS-CoV genome for the construction of p15A-amp-SADS-CoV-WT-F11-F14-HDVrz-SV40polyA |
| SADS-CoV-F13-R | CTTGTTTAACTGCAGCAAC |  |
| SADS-CoV-F14-F | GCCGTCACAGTCTGTTGACATTG | Linear fragment 14 of SADS-CoV genome for the construction of p15A-amp-SADS-CoV-WT-F11-F14-HDVrz-SV40polyA |
| SADS-CoV-F14-R | TGTGTATCACTGTCAAAGAATC |  |
| **pBeloBAC11-cm-CI-SADS-CoV-WT-F1-F14-HDVrz-SV40polyA** | | |
| BAC-cm-SADS-CoV-F | GGAGATGTGGGAGGTTTTTTAAAGCAAGTAAAACCTCTACAAATGTGGTAGCGGCCGCGGGCCCCTCTAGAGTCGACCTGCAGG | Linear fragment BAC-cm for the construction of pBeloBAC11-cm-CI-SADS-CoV-WT-F1-F14-HDVrz-SV40polyA |
| BAC-cm-SADS-CoV-R | CTATATAACCAATGAATAATATGGCTAATGGCCAATATTGAGCGGCCGCGGGCCCCGGGTACCGAGCTCGAATTC |  |
| **pCI-amp-SADS-CoV-N** | | |
| pCI-amp-F | TCTTGAGGAGGAGGTTGAGATGGTGGATGAGATTATTAATTAGttccctttagtgagggttaatg | Linear fragment pCI-amp for the construction of pCI-amp-SADS-CoV-N |
| pCI-amp-R | CCGCCTGTTCAACAGCGTCACCCCAATTAACAGTGGCCATATTCGTTTAGTcctatagtgagtcg |  |
| SADS-CoV-N-F | ATGGCCACTGTTAATTGG | N gene linear fragment of SADS-CoV genome for the construction of pCI-amp-SADS-CoV-N |
| SADS-CoV-N-R | ATTAATAATCTCATCCACCATCT |  |
| **pBeloBAC11-cm-CI-SADS-CoV-WT-F1-F14-HDVrz-SV40polyA-ΔNS3a** | | |
| ΔNS3a-amp-ccdB(knockout)-F | ACTTCAATCATACGAGATTGAAAAGGTCCACGTCCAATAAGAAACTCAACTAAACTATGTTTCTGAAGATTGTTGAGGATGGCGCGCCTCAAGAAGATCCTTTGATCT | For NS3a gene deletion. The homology arms were designed using the SADS-CoV sequence; primer ΔNS3a-amp-ccdB(knockout)-F carries both left and right homology arm sequences. |
| ΔNS3a-amp-ccdB(knockout)-R | ATCCTCAACAATCTTCAGAAACATAGTTTAGTTGAGTTTCGGCGCGCCGGTGTGGTAGCTCGCGTATT |  |
| **pBeloBAC11-SADS-CoV-ΔNS3a-PEDV(COE+S1D)** | | |
| ΔNS3a-ampccdB(knockin)-F | TAAACTTCAATCATACGAGATTGAAAAGGTCCACGTCCAATAATGGGCGCGCCTCAAGAAGATCCTTTGATCT | For NS3a gene deletion. The homology arms were designed using the SADS-CoV sequence. |
| ΔNS3a-ampccdB(knockin)-R | CCATCATCCTCAACAATCTTCAGAAACATAGTTTAGTTGAGTTTCTCAGGCGCGCCGGTGTGGTAGCTCGCGTATT |  |
| ΔNS3a-PEDV-F(WYBD) | TAAACTTCAATCATACGAGATTGAAAAGGTCCACGTCCAATAATGATTTCTTTTGTTACTCTGCCATC | Linear fragment ΔNS3a-PEDV(COE+S1D) for the construction of pBeloBAC11-cm-CI-SADS-CoV-WT-F1-F14-HDVrz-SV40polyA. The homology arms were designed using the SADS-CoV sequence. |
| ΔNS3a-PEDV-R(WYBD) | CCATCATCCTCAACAATCTTCAGAAACATAGTTTAGTTGAGTTTCTCAACTAAAGTTGGTGGGAATACTAAT |  |
| **pBeloBAC11-SADS-CoV-ΔNS3a-PEDV(COE+S1D)-Co1** | | |
| PEDV-Co1-R1 | GGTAATGGTGAACGAGCTGGTAATTGATGAAATGATGAACCACTAAAGTTGGTGGGAATACTAAT | Linear fragment ΔNS3a-PEDV(COE+S1D)-Co1 for the construction of pBeloBAC11-SADS-CoV-ΔNS3a-PEDV(COE+S1D)-Co1. The first round PCR primers were ΔNS3a-PEDV-F(WYBD) (forward) and PEDV-Co1-R1 (reverse); the second round PCR primers were ΔNS3a-PEDV-F(WYBD) (forward) and PEDV-Co1-R2 (reverse). The homology arms were designed using the SADS-CoV sequence. |
| PEDV-Co1-R2 | CATCCTCAACAATCTTCAGAAACATAGTTTAGTTGAGTTTCTCATGGTAATGGTGAACGAGCTGG |  |
| **pBeloBAC11-SADS-CoV-ΔNS3a-PEDV(COE+S1D)-DCpep** | | |
| PEDV-DCpep-R1 | GGACGTTGTGGTGTTGAATGATATGATGGATAAAATGAACCACTAAAGTTGGTGGGAATACTAAT | Linear fragment ΔNS3a-PEDV(COE+S1D)-DCpep for the construction of pBeloBAC11-SADS-CoV-ΔNS3a-PEDV(COE+S1D)-DCpep. The first round PCR primers were ΔNS3a-PEDV-F(WYBD) (forward) and PEDV-DCpep-R1 (reverse); the second round PCR primers were ΔNS3a-PEDV-F(WYBD) (forward) and PEDV-DCpep-R2 (reverse). The homology arms were designed using the SADS-CoV sequence. |
| PEDV-DCpep-R2 | CATCCTCAACAATCTTCAGAAACATAGTTTAGTTGAGTTTCTCATGGACGTTGTGGTGTTGAATG |  |
| **pBeloBAC11-SADS-CoV-ΔNS3a-PEDV(COE+S1D)-Co1-DCpep** | | |
| PEDV-Co1-DCpep-R1 | TGGATAAAATGAGCCACCGCCACCTGGTAATGGTGAACGAGCTGGTAATTGATGAAATGATGAACCACTAAAGTTGGTGGGAATACTAAT | Linear fragment ΔNS3a-PEDV(COE+S1D)-Co1-DCpep for the construction of pBeloBAC11-SADS-CoV-ΔNS3a-PEDV(COE+S1D)-Co1-DCpep. The first round PCR primers were ΔNS3a-PEDV-F(WYBD) (forward) and PEDV-Co1-DCpep-R1 (reverse); the second round PCR primers were ΔNS3a-PEDV-F(WYBD) (forward) and PEDV-Co1-DCpep-R2 (reverse). The homology arms were designed using the SADS-CoV sequence. |
| PEDV-Co1-DCpep-R2 | ATCCTCAACAATCTTCAGAAACATAGTTTAGTTGAGTTTCTCATGGACGTTGTGGTGTTGAATGATATGATGGATAAAATGAGCCACCGC |  |

**Table S2.** Primer sequences for PCR confirmation of insertions in SADS-CoV NS3a site.

| Primer name | Primer sequence (5’-3’) |
| --- | --- |
| SADS-A25-F | GCTCATAACGTCTCTAACATGCG |
| SADS-A25-R | GCATGGTTCAACTTCCATATAAGAC |

**Table S3.** Fluorescence quantitation detection primers and probe sequences for SADS-CoV and PEDV.

| Primer name | Primer sequence (5’-3’) |
| --- | --- |
| SADS-qF | CTGACTGTTGTTGAGGTTAC |
| SADS-qR | TCTGCCAAAGCTTGTTTAAC |
| SADS-probe | TCACAGTCTCGTTCTCGCAATCA |
| PEDV-N-F2 | AGGACCAGCAAATTGGATACT |
| PEDV-N-R2 | CGAGTCCTATAGCGGAGGT |
| PEDV-probe | ATTGGCATTTCTACTACCTCGGAACAGG |
